# A High Throughput Ambient Mass Spectrometric Approach to Species Identification and Classification from Chemical Fingerprint Signatures

**DOI:** 10.1038/srep11520

**Published:** 2015-07-09

**Authors:** Rabi A. Musah, Edgard O. Espinoza, Robert B. Cody, Ashton D. Lesiak, Earl D. Christensen, Hannah E. Moore, Simin Maleknia, Falko P. Drijfhout

**Affiliations:** 1 Department of Chemistry, University at Albany, State University of New York, 1400 Washington Avenue, Albany, NY 12222 USA; 2 U.S. National Fish and Wildlife Forensics Laboratory, 1490 East Main Street, Ashland, OR, 97520-1310, USA; 3 JEOL USA Inc., 11 Dearborn Road, Peabody, MA 01960 USA; 4 National Renewable Energy Laboratory, 15013 Denver West Parkway, MS-1634, Golden, CO 80401 USA; 5 Department of Chemical Ecology, School of Physical and Geographical Science, Keele University, Keele ST5 5BG, UK; 6 School of Biological, Earth and Environmental Sciences, University of New South Wales, Sydney, Australia

## Abstract

A high throughput method for species identification and classification through chemometric processing of direct analysis in real time (DART) mass spectrometry-derived fingerprint signatures has been developed. The method entails introduction of samples to the open air space between the DART ion source and the mass spectrometer inlet, with the entire observed mass spectral fingerprint subjected to unsupervised hierarchical clustering processing. A range of both polar and non-polar chemotypes are instantaneously detected. The result is identification and species level classification based on the entire DART-MS spectrum. Here, we illustrate how the method can be used to: (1) distinguish between endangered woods regulated by the Convention for the International Trade of Endangered Flora and Fauna (CITES) treaty; (2) assess the origin and by extension the properties of biodiesel feedstocks; (3) determine insect species from analysis of puparial casings; (4) distinguish between psychoactive plants products; and (5) differentiate between *Eucalyptus* species. An advantage of the hierarchical clustering approach to processing of the DART-MS derived fingerprint is that it shows both similarities and differences between species based on their chemotypes. Furthermore, full knowledge of the identities of the constituents contained within the small molecule profile of analyzed samples is not required.

One of the manifestations of the genetic differences that distinguish one species from another is in the profile of constitutively present small molecules they contain, also known as the metabolome. Since the small-molecule profile of an organism ultimately reflects the genes that distinguish it, the information content of the metabolome might be just as well suited to genomic fingerprinting and assessment of genetic relatedness between species as the genomes themselves. There are several reasons why it would be useful to be able to accurately correlate the signature of small molecules observed within an organism to its overall systems biology. The observation of the composite of small-molecule biomarkers could provide a real-time view of gene expression activity, enable the monitoring of the status of cellular transcriptomes and proteomes, provide a means of assessing the evolutionary history of organisms, and provide an avenue for the rapid monitoring of the success of gene knockouts and knockdowns, among other uses. Although these applications can be accomplished by phylogenetic methods, the paucity of mapped and/or annotated genes for the vast majority of fauna and flora in existence makes this approach impossible for all but a select group of mostly model systems.

Convenient characterization of the defining features of an organism’s real-time chemical portrait for the purpose of species classification has been hampered by several factors. These include: (1) the difficulty of acquiring a comprehensive small-molecule chemical map of an organism or its parts in real time; (2) the time-consuming nature of metabolome profiling by conventional methods; (3) the challenge of obtaining a faithful and consistent representation of defining chemical components or chemical component ratios that is divorced from biases or artifacts introduced by sample processing steps; and (4) distinguishing between chemicals that define a species and those that do not provide discriminatory information. However, the advent within the last decade of ambient ionization mass spectrometric methods that feature instantaneous real-time detection of fairly comprehensive small molecule profiles of matter in its native form, has the potential to revolutionize and simplify metabolome- and/or chemical fingerprint-based species characterization by circumventing to a large extent the aforementioned deficiencies of conventional methods. Additionally, the utilization of the comprehensive mass spectrometry-derived fingerprint, rather than a subset of small-molecule biomarkers, provides the opportunity to subject an entire dataset to multivariate statistical analysis to aid in species classification, as well as processing of the data through hierarchical clustering in order to assess genetic relatedness and distinguish between species.

Direct Analysis in Real Time (DART®)^1^ is one of the most common of the new mass spectrometric “ambient ionization” sources^2^ and was the first such source to be introduced as a commercial product. Following an early application note on the use of DART to analyze the flavor components and polyphenols in the leaves of two different basil cultivars^3^, there have been several reports on the application of DART to species biomarker identification. Fatty acid profiles measured by DART for different bacterial species have been shown to be distinct and reproducible^4^, volatiles release from Eucalypts of different species have been shown to be unique^5^,^6^, differentiation between red oak (*Quercus rubra*) and white oak (*Q. alba*)^7^ by DART has been demonstrated, identification of printing and writing papers^8^ based on chemical profile differences has been shown, and the identification of *Piper betel* cultivars^9^, ambiguous cubeb fruit^10^ and varieties of the psychoactive plant *Mitragyna speciosa* (“Kratom”)^11^ have all been demonstrated utilizing ambient DART mass spectrometry. The U.S. Fish and Wildlife Services Forensic Laboratory has also used DART-MS to distinguish between species of *Dalbergia*^12^ and agarwood^12^. Each of the aforementioned reports relied on visual examination of mass spectra or their corresponding heat maps for selection of *m/z* features that were then used in chemometric-based approaches including the unsupervised learning methods Principal Component Analysis (PCA) and Partial Least Squares Discriminant Analysis (PLS), and the supervised learning methods Linear Discriminant Analysis (LDA) and Kernel Discriminant Analysis (KDA), for distinguishing between species within a single genus. Successful application of these methods requires careful albeit *a priori* selection of the features (mass spectral peaks) that differentiate between species.

By combining mass spectrometric heat maps and chemometric protocols, we illustrate a high throughput method by which DART-MS-derived chemical signature profiles can be subjected to cluster analysis to not only distinguish between species, but provide information on genetic relations. An advantage of the hierarchical clustering approach to the processing of DART-derived fingerprint information is that it shows both similarities and differences between species based on their chemotypes as determined from DART-MS data. In contrast, previously described chemometric methods show only differences between classes, but do not indicate which classes have similar chemotypes. Furthermore, full knowledge of the identities of the constituents contained within the small molecule profile of the sample being analyzed is not required. The method is robust and rapid and the results are consistent. Here, we showcase several applications although these are by no means exhaustive and numerous other possibilities exist. In this work, we demonstrate how the method can be used to: (1) distinguish between endangered woods regulated by the Convention for the International Trade of Endangered Flora and Fauna (CITES) treaty; (2) assess the origin and by extension the properties of biodiesel feedstocks; (3) determine insect species from analysis of puparial casings; (4) identify psychoactive plant products; and (5) differentiate between Eucalyptus species.

## Results

### Detection of Illegally Traded Endangered Species in the Genus Dalbergia

The convention for the international trade of endangered flora and fauna (CITES) which is enforced under the Endangered Species Act (ESA), bans the trade of tree species whose harvest has been deemed unsustainable. Visual inspection of harvested plant products in which leaves, flowers and other characteristic features have been retained can enable definitive identification of endangered species. However, since timber and sawn boards generally lack diagnostic morphological features, identification of wood products has historically relied on anatomical or chemical features associated with the hardwood. This process is laborious, time-consuming and can be prone to error.

The aforementioned challenge is further exacerbated not only by the plethora of colloquial terms used within the timber trade community for any one tree species, but also by the common use of a single name to refer to multiple species. For example, “*Dalbergia granadillo* Pittier” is a tree species of rosewood endemic to Mexico and northern Central America, whose common name is “granadillo”. However, this same name has been used to describe *Dalbergia retusa* (Leguminosae family), and several Fabaceae family trees including *Platymiscium yucatanum, Caesalpinia echinata,* and *C. platyloba.* Furthermore, *D. retusa* has a large number of synonyms including *Amerimnon lineatum, A. retusum, D. cuscatlantica, D. hypoleuca, D. lineata, D. pacifica, D. retusa var. hypoleuca,* and *D. retusa* var. *lineata*, with many xylarium collections still using historical nomenclature. The institution of correct *Dalbergia* classifications and nomenclature and the identification of illegally traded endangered *Dalbergia* species, has been stymied by the absence of a rapid and consistent mechanism by which to distinguish between species, and routinely detect those that are banned. In this work the chemical profile derived from a DART ion source coupled to a high-resolution time-of-flight (TOF) mass spectrometer was used to rapidly and consistently identify and distinguish between *Dalbergia* species.

**Figure 1:**
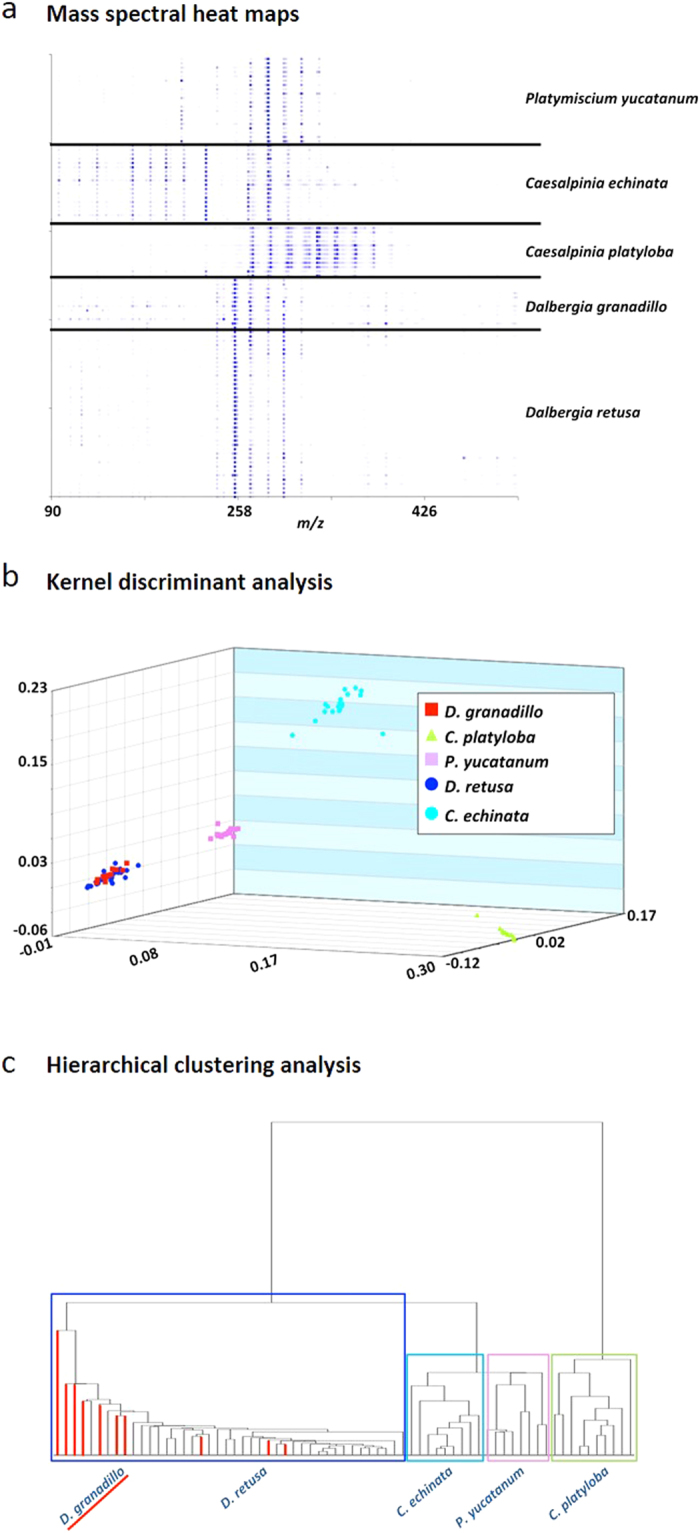
DART-TOF mass spectral heat maps, kernel discriminant analysis (KDA) and hierarchical clustering analysis results derived from the DART-TOF mass spectra of *Dalbergia* wood species. Panel **a**: DART-TOF mass spectra rendered as heat maps of the indicated species; Panel **b**: KDA based on 104 feature masses; 3 principal components accounted for 84.51 % of the variance. The LOOCV was 98.98%. Panel **c**: hierarchical clustering analysis dendrogram created from mass spectral heat map data, showing species classifications of the analyzed *Dalbergia* species woods. The leaves highlighted in red indicate *D. grandillo* specimens which cluster with *D. retusa*.

We proceeded on the premise that the rare *D. granadillo* was a synonymous species to other less cryptic taxa. Because the known curated xylarium reference samples of *D. granadillo* are extremely rare (n = 11 worldwide), we decided to first compare the known xylarium-authenticated samples of *D. granadillo* hardwood by DART-TOF-MS to determine whether they exhibited similar chemical fingerprints. The results were then contrasted with the DART-TOF-MS spectra of five species of timber from a variety of sources that have been described by the common name of “granadillo” (i.e. *D. granadillo*, *D. retusa*, *P. yucatanum, C. echinata*, and *C. platyloba*—Supplementary Table 1). The mass spectra generated (Supplementary Figure 1) were rendered as heat maps using the Mass Mountaineer software suite. Supplementary Tables 2a-c show the corresponding measured *m/z* values and their abundances, and Fig. 1a illustrates the mass spectral heat maps. The results show that *D. retusa* and *D. granadillo* have similar compounds present in roughly the same relative amounts as indicated by the similar intensities of the indicated colors in the heat maps. The other three species show different and distinctive diagnostic ion patterns. The high-resolution masses of several of the molecules detected were consistent with those of compounds previously reported to be present in *Dalbergia*^13^,^14^.

Figure 1b is a graphical representation of the Kernel discriminant analysis (KDA) plot generated using 104 feature masses ranging from *m/z* 107.037 – *m/z* 527.155 from a training set of 102 spectra. The plot shows that *D. retusa* and *D. granadillo* form a single cluster that cannot be differentiated using KDA. The leave-one-out cross validation (LOOCV) for the KDA classification model analysis was fairly poor at 64.29%, reflecting the fact that these *Dalbergia* species could not be separated. The clustering of *D. retusa* and *D. granadillo* is supportive of our hypothesis that both represent one and the same species and therefore should be described by a single name. Indeed, when *D. retusa* and *D. granadillo* were joined into a single group under one name (*D. retusa*), the LOOCV of the KDA model rose to 98.98%. Thus, our observations support the premise that from the chemical profile of the heartwood, *D. granadillo* cannot be distinguished from *D. retusa* and that *D. granadillo* and *D. retusa* may be synonymous. Interestingly, we found that when the heat map data were imported into a third-party hierarchical clustering program such as Cluster 3.0, the resulting dendrogram classified the various *Dalbergia* samples according to species and illustrated their genetic relatedness. Cluster analysis was performed using uncentered correlation of 436 variables of the spectral data, and a typical result is shown in Fig. 1c. The leaves highlighted in red in Fig. 1c are *D. granadillo* specimens. The dendrogram shows that the *D. granadillo* clusters with the *D. retusa* samples, supporting the hypothesis that both represent the same species.

### Inferring the Phylogeny of Biodiesel Feedstocks From Fatty Acid Methyl Ester (FAME) Profiles

Biodiesel is a renewable fuel derived from vegetable oils or animal fats by transesterification of triglycerides with an alcohol, generally methanol, in the presence of a catalyst^15^. The resulting mixture of fatty acid methyl esters (FAMEs) can be used to fuel diesel engines and is most often blended with petroleum diesel. The amount of biodiesel produced in the U.S. has increased significantly in recent years. In 2010 production was just over 300 million gallons, which increased to nearly 1 billion gallons in 2011. In 2013, production reached over 1.3 billion gallons^16^. Increased production and utilization of biodiesel has intensified interest in the properties of this fuel and how these properties impact engines and infrastructure. The feedstock from which biodiesel is derived determines many of the properties of the fuel. These properties are directly related to fatty acid makeup of different oil sources^17^. Desired properties such as cold weather operability and resistance to autoxidation are influenced by the acyl chain length and degree of unsaturation of the fatty acids in the feedstock^18^,^19^. If the feedstock used to manufacture a biodiesel is unknown to the user, the source may be determined from the FAME profile of the product if the unique fatty acid distribution of the source oil is known^20^. FAME profiling is commonly achieved with gas chromatography^21^. This analysis can be time consuming, particularly if a high degree of resolution is required to isolate FAMEs in more complex samples.

**Figure 2:**
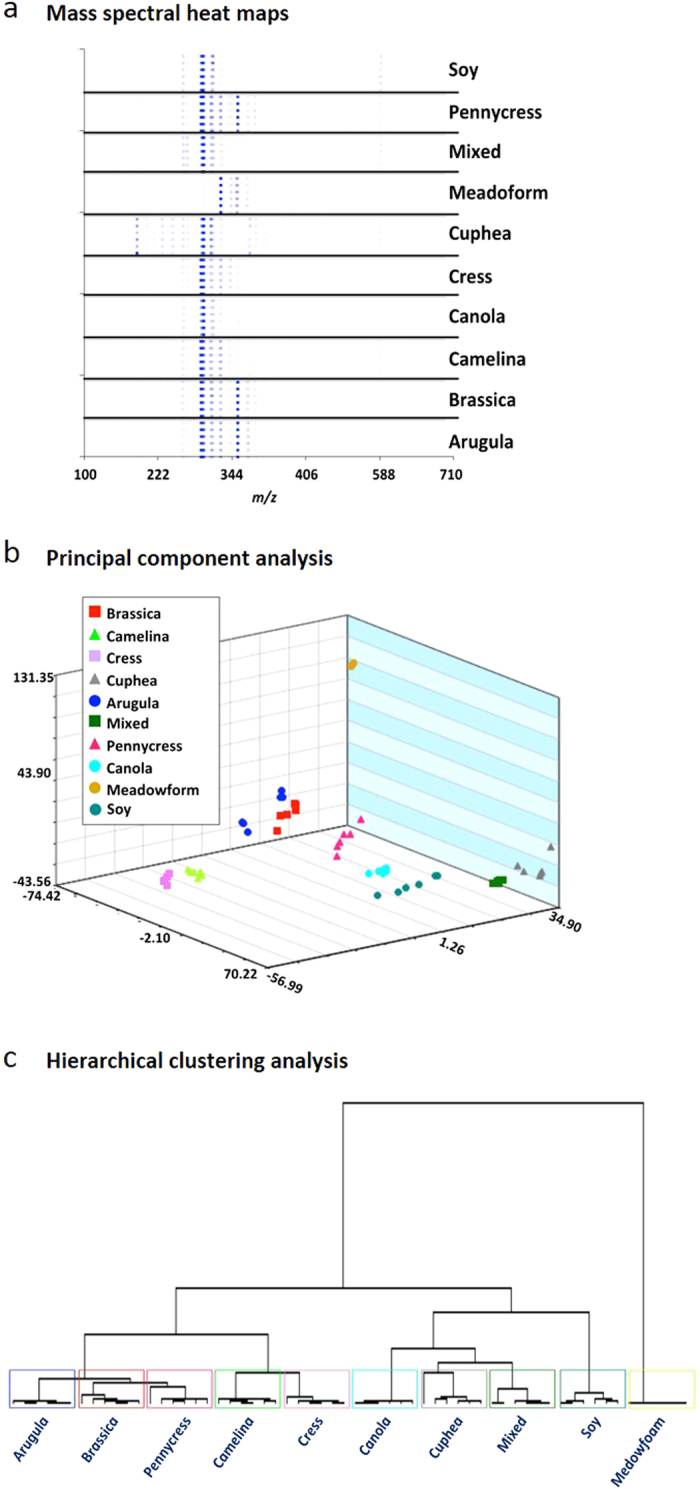
DART-TOF mass spectral heat maps, principal component analysis (PCA) and hierarchical clustering analysis results derived from the DART-TOF mass spectra of biodiesel feedstocks solubilized in hexane. Panel **a**: DART-TOF mass spectra rendered as heat maps of the indicated feedstocks; Panel **b**: PCA was based on sixteen feature masses; 3 principal components accounted for 73.4% of the variance and the LOOCV was 98.3%. Panel **c**: hierarchical clustering analysis dendrogram created from mass spectral heat map data, showing species-level classifications of the analyzed feedstocks.

We determined that positive-ion DART can be used to rapidly determine the FAME profile of biodiesel, allowing for quick source and properties identification. The biodiesel samples utilized in this study included the most commonly used feedstocks in the United States^22^. Arugula (*Eruca sativa*), Brassica (*Brassica juncea*), Field Pennycress (*Thlaspi arvense*), Cress (*Lepidium sativum*), Camelina (*Camelina sativa*), Meadowfoam (*Limnanthes alba*), and Cuphea (*Cuphea lanceolata*) seed oils were provided by the USDA National Center for Agricultural Utilization Research. The DART mass spectra (Supplementary Figure 2) obtained for hexane solutions of the aforementioned ten biodiesel feedstocks were dominated by both saturated and unsaturated FAMEs ranging in size from 11–23 carbons (Supplementary Table 3). Hexane was used to dilute the samples because they proved to be too concentrated in their native form. The most abundant species in the majority of feedstocks (i.e. Brassica, Camelina, Canola, Cuphea, Pennycress and Soy) were C_19_ FAMEs (derived from C_18_ fatty acids). Nevertheless, the mass spectra were consistent for samples within the same species, but very clearly different between species (Supplementary Figure 2). Fifteen feature masses were used for principal component analysis (Fig. 2b). Species level clustering was observed in the covariance PCA plot with five principal components accounting for 92.6% of the variance and the LOOCV was 98.33%. Subjection of the corresponding mass spectral heat maps (Fig. 2a) to hierarchical clustering analysis showed that each species was clearly separated from the others (Fig. 2c). All members of Order *Brassicale* were clustered together with the exception of Canola, which is a cultivar that has been bred to have low erucic acid content. The remaining feedstocks (including the mixed biodiesel) belonging to different orders and/or families, comprised a separate cluster. Interestingly, Meadowfoam (order *Brassicale*, family *Limnanthaceae*) was distinct from all of the other species. These observations illustrate that easily and rapidly acquired feedstock chemical profile information can be translated into dendrograms that clearly distinguish between genera to show their evolutionary relationships, and that the data can be generated in a high throughput fashion.

### Fly Species Identification from Insect Puparial Cases

Blowflies (Diptera: Calliphoridae) are important to forensic entomology because they are often the first colonizers of decomposing remains and can offer significant diagnostic information towards calculating an accurate minimum post mortem interval (PMI_min_). Calliphoridae puparial cases are often the only persisting entomological evidence in criminal investigations involving highly decomposed remains^23^. These cases are the empty shells of the last layer of the larval stage (post feeding). Many studies have been published using larvae and pupal stages for PMI estimations^24^,^25^,^26^,^27^,^28^, but much less research has been published on puparial cases and currently, they are rarely used in criminal investigations due to the difficulty in identifying and ageing them. However, in the past decade, some studies have suggested that invaluable information can be extracted from puparial cases and hence, new methods to identify them are being developed^23^,^29^.

When an adult fly emerges from the case it does so from the mouth end, leaving the rest of the case intact. To correctly identify them, the same morphological features used for the pupae are examined (i.e. posterior spiracles, spines, and mouth piece if present). However, with empty cases, these morphological features have often been destroyed during emergence of the adult fly. It is well established that insect cuticular hydrocarbons have characteristic profiles for different species^30^,^31^,^32^,^33^,^34^. The same holds true for the hydrocarbon profiles of insect puparial cases^35^. Therefore, hydrocarbon analysis is advantageous because both young and aged cases retain definitive chemical information due to the stability of the constituent hydrocarbons despite weathering effects.

**Figure 3:**
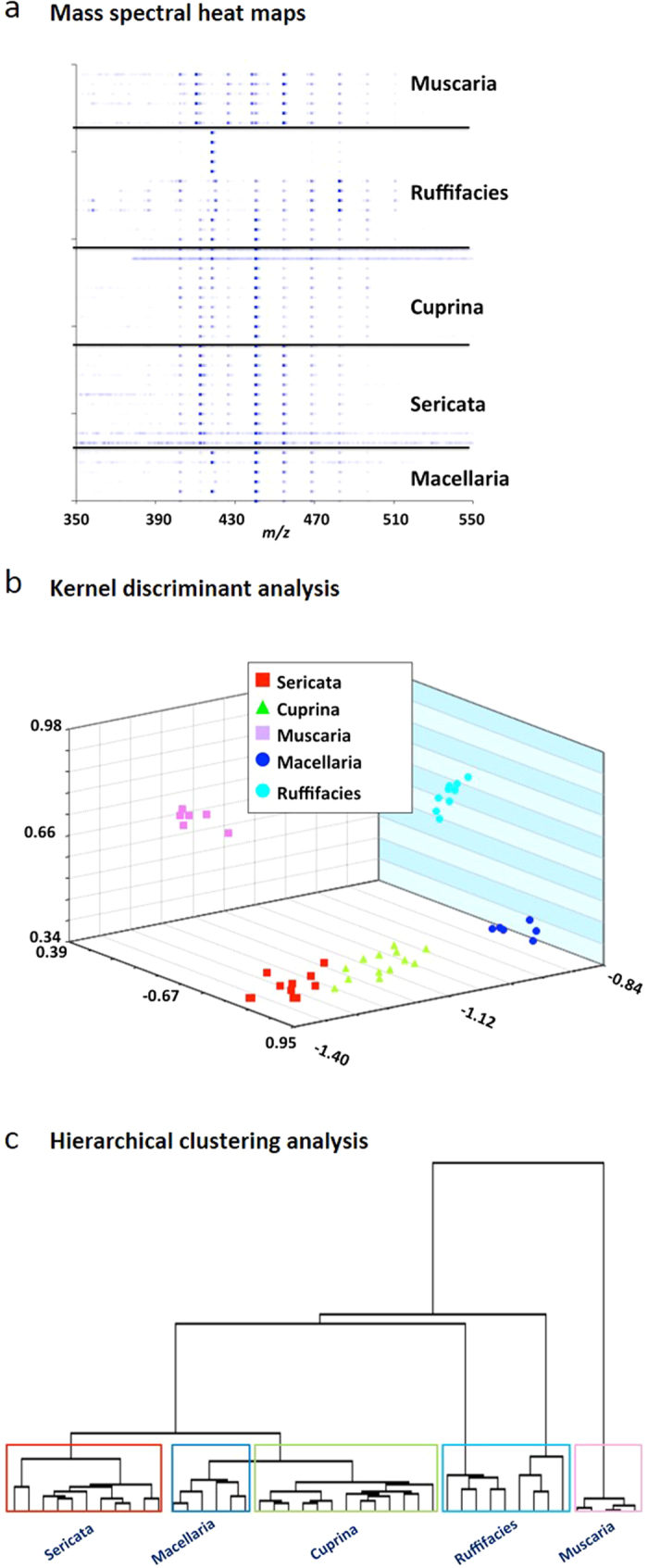
DART-TOF mass spectral heat maps, Kernel discriminant analysis (KDA) and hierarchical clustering analysis results derived from the DART-TOF mass spectra of hexane extracts of puparial cases of *C*. *rufifacies*, *L. sericata*, *L. Cuprina,* and *C. macellaria* and *M. domestica.* Panel **a**: DART-TOF mass spectra rendered as heat maps; Panel **b**: Kernel discriminant analysis (KDA) was based on 10 feature values. Five principal components accounted for 96% of the variance. The LOOCV was 100%. Panel **c**: hierarchical clustering analysis dendrogram created from mass spectral heat map data, showing species classifications of the analyzed puparial cases.

Positive-ion DART-MS was previously used to analyze the unsaturated cuticular hydrocarbons of awake behaving fruit flies (*Drosophila melanogaster*)^36^. However, this form of analysis does not give clear, unambiguous mass spectra for saturated alkanes. Recently, we reported that large polarizable alkanes, lipids and alcohols can be detected as O_2_^−^ adducts ([M + O_2_]^−^) by aspirating sample solutions directly into the mass spectrometer atmospheric pressure orifice in the presence of the O_2_^−^ generated by the DART ion source^37^. We applied this technique to our analyses. The mass spectra typically observed are presented in Supplementary Figure 3 and the heat map renderings of these spectra are shown in Fig. 3a. The detected C_27_-C_34_ alkanes that were used as the basis for multivariate statistical analysis by supervised methods were easily observed as O_2_^−^ adducts (see mass spectral peak assignments and molecule abundances in Supplementary Table 4). All species are distinctly separated within the PCA plot, demonstrating that their profiles have unique chemical differences (Supplementary Figure 3). However, there is less separation between *L. cuprina* and *L. sericata*. This is likely because they are from the same genus (*Lucilia*) and therefore their profiles share more similarities compared to the other species. It should be noted that this method does not distinguish between hydrocarbon isomers or provide any information about branching. Nevertheless, the results confirm that the hydrocarbon profiles measured by DART-MS clearly enable distinctions between species to be made.

A training set comprised of blowfly puparial case hexane extract mass spectra (i.e. *Chrysomya rufifacies*, *Lucilia sericata*, *L. cuprina*, and *Cochliomyia macellaria*) as well as spectra of puparial case extracts of the common house fly (*Musca domestica*) was created. Feature masses from these spectra were used for KDA, with the results featured in Fig. 3b. Excellent separation between the five different insect types was observed and LOOCV gave 100% correct identification for all samples. Five sets of puparial cases labeled “A” though “E” that were provided as blind samples were then analyzed. Samples A, B, C and D were correctly identified as *L. sericata, C. rufifacies, L. cuprina,* and *C. macellaria* respectively. Sample E gave a distinctly different profile. It was later revealed that it represented puparial cases for the common housefly, *Musca domestica*.

Although the mass spectral data for Sample B correctly clustered with that of *C. rufifacies*, it differed somewhat from the standard *C. rufifacies* samples measured one month earlier. This is illustrated in a comparison of the Sample B spectrum (Supplementary Figure 4) with that of the spectrum obtained for *C. rufifacies* (Supplementary Figure 3). The reason for this difference is not clear, but preliminary observations indicate that the alkane profiles for puparial cases of a given species may vary with age^35^. Although this difference is evident in the PCA plot (Fig. 3b), hierarchical clustering analysis of the mass spectral datasets that were rendered as heat maps showed the B samples and the *C. rufifacies* standards clustering together (Fig. 3c). The DART-MS derived small molecule fingerprint of a puparial case as a function of its age is currently being investigated by the authors, as a correlation between the two could potentially serve as a tool in post mortem investigations.

### Species Identification From Seeds of Plants Containing Belladonna Alkaloids

The genus *Datura* contains multiple species of ornamental flowering plants of horticultural importance. They are a well-known source of belladonna alkaloids including scopolamine and atropine, whose hallucinogenic and narcotic properties have been exploited in traditional religious rituals, herbal and mainstream medicine, and more recently in recreational drug abuse using its seeds^38^. It is often difficult to distinguish *Datura* species due to similarities in the appearance of both their seeds and their aerial parts, and because their morphological features can vary depending on where the plants are grown^39^. *Datura* plants also often bear resemblance to those in the *Brugmansia* genus, and this has led to the misidentification of some genus *Brugmansia* plants as belonging to the *Datura* genus and vice versa, as well as misidentification of species within the *Datura* genus^39^. An additional challenge is that the morphological features that allow the plants to be distinguished, most notably the flowers and fruits, take months to years to appear, making species differentiation a long-term project. Furthermore, from a chemical profiling standpoint, the belladonna alkaloids found in *Datura* species also appear in *Brugmansia* and *Hyocyamus* species, making identification of plant material based on the presence of belladonna alkaloid biomarkers alone indeterminate. All of the aforementioned species are members of the large group of non-model plants that are poorly annotated and whose genomes have not been mapped, making phylogenetic species identification impossible. Here, direct analysis in real time-mass spectrometry (DART-MS) and hierarchical clustering analysis tools were applied to the seeds. The approach provided a rapid high throughput and viable method to test seeds directly for identification purposes, as well as for species differentiation and classification by cluster analysis.

**Figure 4:**
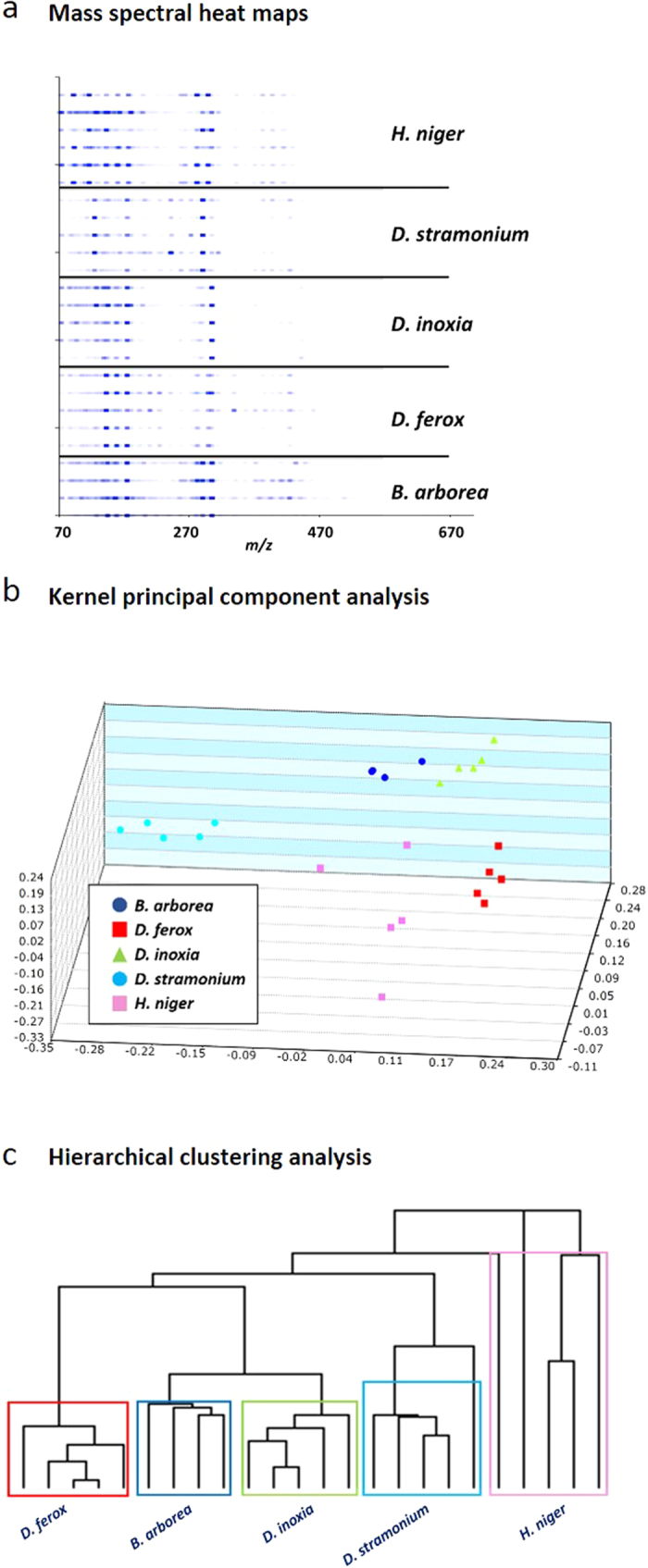
DART-TOF mass spectral heat maps, Kernel principal component analysis (KPCA) and hierarchical clustering analysis results derived from the DART-TOF mass spectra of *B. arborea*, *D. ferox*, *D. inoxia*, *D. stramonium* and *H. niger* seeds. Panel **a**: DART-TOF mass spectra of the five species rendered as heat maps; Panel **b**: Kernel principal component analysis (KPCA) based on 31 feature masses. Five principal components accounted for 64% of the variance. The LOOCV was 96%; Panel **c**: hierarchical clustering analysis dendrogram created from mass spectral heat map data, showing species classifications of the analyzed plant seeds.

Of the known *Datura* species, we used *D. ferox*, *D. stramonium* and *D. inoxia*, as these are commonly abused seeds. Since the belladonna alkaloids that serve as biomarkers for *Datura* species are also present in *Brugmansia* and *Hyocyamus* seeds, both were also analyzed to assess whether they could be distinguished as species unique from *Datura*. Figure 4a shows the mass spectral profiles of all five species, done in replicates of 3-5, rendered as heat maps. The corresponding raw mass spectra and the peak abundances are presented in Supplementary Figure 5 and Supplementary Tables 5a-5e respectively. The high resolution data revealed that several of the detected molecules had molecular formulas consistent with molecules that have been observed in *Datura* spp. such as tropine, scopoline, dihydroxytropane, hexose sugars, scopolamine, 3-tigloyloxy-6,7-dihydroxytropane, vanillin, linoleic acid and oleic acid (see Supplementary Tables 5b-5d)^40^,^41^,^42^. By visual inspection it was apparent that the mass spectra of each species were quite unique, even for the three *Datura* species. A total of 31 feature masses representing diagnostic peaks were used as a training set for the Kernel principal component analysis (KPCA). The results are shown in Fig. 4b. Each of the species was well clustered and could be distinguished from the others. Nevertheless, three principal components accounted for only 40% of the variance and increasing the number of principal components to 5 accounted for only 64% of the total variance. However, the LOOCV was 96%. The power of the chemical fingerprint signatures in permitting species differentiation and classification was demonstrated when the heat map data were imported into Cluster 3.0. Processing of the data in this manner furnished a dendrogram in which each of the seeds of the same species fell within clades that were representative of species classifications based on morphological feature differences, and were clearly distinct from one another (Fig. 4c).

### Species Differentiation of Eucalypts From Mass Spectrometry-derived Tissue-dependent Chemical Fingerprints

The *Eucalyptus* genus covers a diverse range of flowing trees and shrubs with more than 700 species that are broadly distributed throughout the Americas, Australia, Africa, and Europe. They are commonly known as “gum trees” because of the distinct and pleasant volatile exudate that is produced in response to a tissue breach. Many species have attracted global attention as a source for fragrance oils, biofuels, a fast-growing wood source and other commercial applications^43^.

DART-MS profiling of eucalypt species was previously selected as a facile method to classify temperature-dependent emissions of volatile organic compounds (VOCs) for their atmospheric contributions in relation to changing climates and global warming, and to better estimate the range of biogenic pollutants released into the atmosphere during wildfires^5^,^6^. In that work, VOCs from stems and leaves of several eucalypts including *E. cinerea*, *E. citriodora*, *E. nicholii* and *E. sideroxylon* were identified. A wide range of compounds from simple organics (i.e. methanol and acetone) to a series of monoterpenes (i.e. pinene, camphene, cymene, eucalyptol) common to many plant species, as well as less abundant sesquiterpenes and flavonoids, were detected. This was achieved by stepwise adjustment of the DART helium gas temperature from 50 to 100 to 200 and to 300 °C, which enabled direct evaporation of compounds up to the onset of pyrolysis of plant fibres (i.e. cellulose and lignin). The identification of compounds was facilitated by correlating the observed high resolution accurate mass data to plant library compounds, and further matching their theoretical and experimental isotopic distributions.

**Figure 5:**
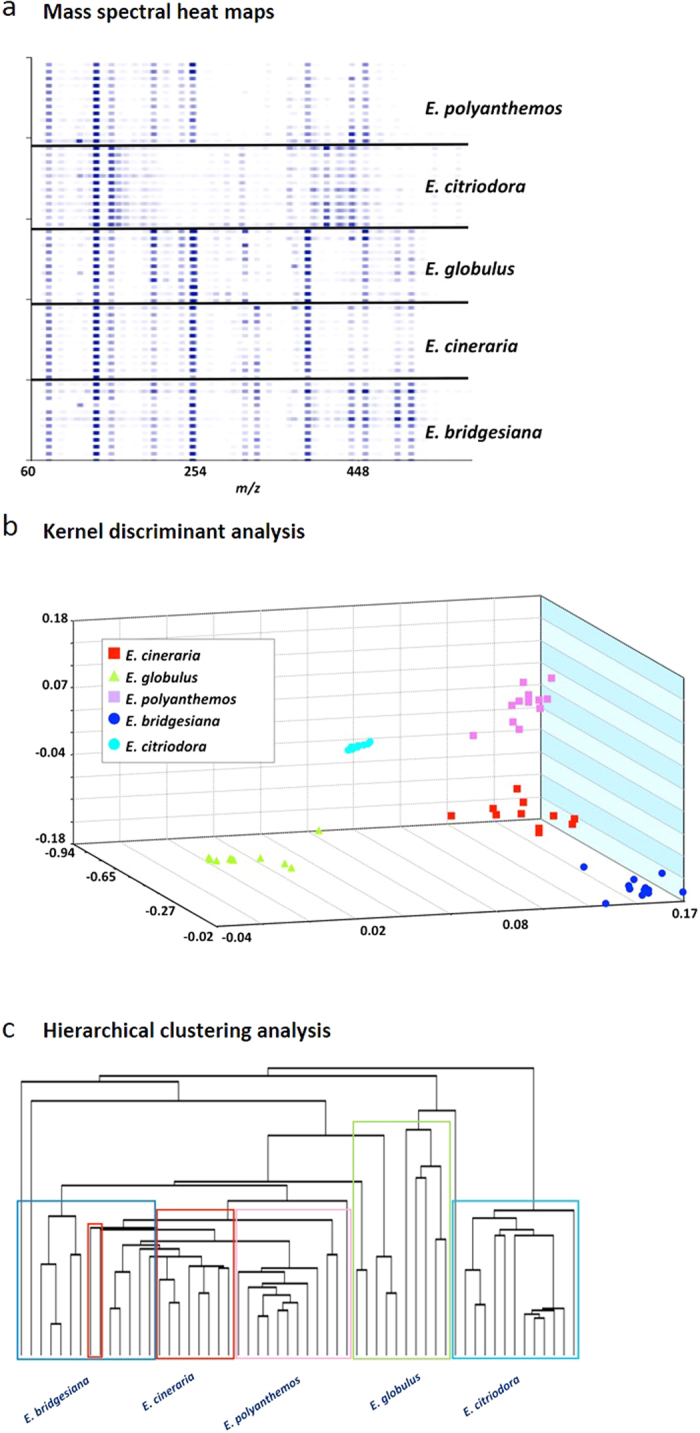
DART-TOF mass spectral heat maps, Kernel discriminant analysis (KDA) and hierarchical clustering analysis results derived from the DART-TOF mass spectra of Eucalypt species leaves. Panel **a**: DART-TOF mass spectra of the five species rendered as heat maps; Panel **b**: KDA based on fifteen feature masses. Three principal components accounted for 93% of the variance. The LOOCV was 82.7%; Panel **c**: hierarchical clustering analysis dendrogram created from mass spectral heat map data, showing species-level classifications of the analyzed plant leaves.

In the current work the initial VOC temperature-dependent emission studies have been extended to chemometric-based processing of mass spectral data for species differentiation. DART-MS analyses of leaf samples at a fixed temperature of 300 °C for several eucalypts including *E. bridgesiana*, *E. cinerea*, *E. globulus*, *E. citriodora* and *E. polyanthemos* was conducted. The observed spectra, each of which represents the average of 5 individual spectra, are shown in Supplementary Figure 6, with the corresponding measured *m/z* and peak abundance values presented in Supplementary Tables 6a-6e. The heat map renderings of the spectra are shown in Fig. 5a. The results revealed the presence of a number of chemotypes common to all species including monoterpenes (*m/z* 137, C_10_H_17_) and various sesquiterpenes (*m/z* 205, C_15_H_25_). Several of the detected formulas are consistent with those of compounds isolated from the species (outlined in Supplementary Tables 6a-6e)^44^,^45^,^46^. Although all the species shared most of the dominant ions, they differed primarily in the relative abundance of detected compounds. A total of 15 feature masses representing *m/z* values varying from 155 to 509 were used for KDA. The resulting plot is shown in Fig. 5b. Three principal components accounted for 94% of the observed variance, and the LOOCV was 83%. When the mass spectral heat maps (Fig. 5a) were processed using Cluster 3.0, the resulting dendrogram (Fig. 5c) showed excellent species level discrimination, and none of the data were misclassified, thus demonstrating the robustness of the approach of using the entire mass spectral data set in providing the information needed for species-level distinctions to be made.

## Discussion

Statistical processing of the output of chemical analysis techniques for the purposes of typing and classification is not new. For example, hierarchical cluster analysis of Raman spectroscopic data has been used to classify tree pollens^47^ and genus *Mentha* plants^48^. Multivariate statistical analysis has been applied to gas chromatographic results to classify the geographic origin of cocoa beans^49^, as well as ^1^H NMR data for the analysis of wines^50^. Chemometric discrimination of coffee beans by area of origin has been demonstrated using Fourier transform infrared spectroscopy^51^.

The output of various mass spectrometric methods of small molecule profiling has also been similarly analyzed with varying results. Examples include multivariate statistical analysis of data generated using: 1) Curie Point pyrolysis mass spectrometry for classification of bacteria^52^; (2) paper spray mass spectrometry for determination of the geographic origin of coffee^53^; (3) HPLC-tandem MS of herbal medicines to determine country of origin^54^; (4) Ultraperformance liquid chromatography-time of flight mass spectrometry for classification of wheat lines^55^; (5) LC-MS/MS for the assessment of the utility of using bioactive components as the basis of distinguishing between herbal medicines^56^; (6) RPLC ESI-MS for standardization of *Ginkgo biloba* extracts^57^; (7) direct injection electrospray MS for classification of coffee trees^58^; (8) ion molecule reaction mass spectrometry for bacterial species differentiation^59^; (9) GC- and atmospheric pressure photoionization (APPI) MS for classification of natural resins^60^; (10) GC-GC TOF/MS for characterization and authentication of edible oils^61^, and (11) pyrolysis GC-MS profiling of eucalypt emissions in response to climate change and wildfires^62^, among other examples. The method described here differs from those outlined in the aforementioned studies in that in general, data acquisition is simpler, a broad range of compounds spanning the dielectric constant spectrum can be detected in a single experiment, and the entire information content of the observed DART-MS-derived chemical fingerprints is subjected to unsupervised hierarchical clustering (rather than using a subset of feature masses and/or chromatographic peaks).

Besides DART-MS, desorption electrospray ionization mass spectrometry (DESI-MS) is another ambient ionization mass spectrometry technique that exhibits advantages similar to those noted for DART-MS. However, relatively few studies featuring DESI-MS in metabolome profiling and/or chemical fingerprinting have appeared. Recently, Watrous *et al.*^63^ demonstrated the use of “nanospray” DESI-MS for the *in vivo* metabolic profiling of bacterial colonies directly from a Petri dish. The report further illustrates the power of ambient ionization mass spectrometric methods to rapidly provide unprecedented glimpses of real-time changes in chemical fingerprint profiles in ways that are difficult and/or impossible to accomplish by more conventional methods.

In this report, we show that a variety of chemotypes from a diversity of samples can be readily detected under similar conditions. The high resolution *Dalbergia* species results revealed the presence of several molecules with formulas consistent with those of compounds that have been identified in *Dalbergia* including neoflavonoid quinone derivatives such as the dalbergiones, various isoflavones, guainolide sesquiterpene lactones, auxins such as indole-3-acetic acid, pyrano- and furano-benzenes and diterpenes among many other polar and non-polar small molecules^13^,^14^. Both saturated and unsaturated biodiesel feedstock-derived FAMEs of from 11 to 23 carbons were easily observed in positive ion mode. In analysis of the biofuels, we observed that the biodiesel was most conveniently analyzed by first diluting it with a non-polar solvent such as hexane, in order to make it less viscous. Alkanes and alkenes from 27–34 carbons long were observed as O_2_^−^ adducts in hexane extracts of fly puparial cases, showing distinct variations in profile and abundance as a function of species. Our approach to the analysis of the puparial cases represents the first published application of the O_2_^−^ attachment ionization technique to address an analytical problem. This novel method enabled us to easily detect large polarizable alkanes, lipids and alcohols as [M + O_2_]^−^ adducts, by aspirating sample solutions directly into the mass spectrometer atmospheric pressure orifice in the presence of the O_2_^−^ generated by the DART ion source^37^. The application of this technique necessitated the use of the solvent which, in this case, was hexane. Although these large non-volatile species could have been detected by GC-MS or field desorption, the former method is much slower than DART-MS analysis, while the latter requires introducing the sample into a vacuum on a fragile emitter. Neither approach is as convenient as the DART-MS analysis described here. In analysis of *Datura*, *Brugmansia* and *Hyocyamus* species plants, a range of compounds of varying polarities was observed, as illustrated in Tables 5a-e. Amines, sugars and fatty acids, among hundreds of other compound types, were all detected in seconds in positive as well as negative ion modes. In the case of the Eucalpyts, direct leaf analysis yielded spectra in which the presence of the odiferous mono- and sesquiterpenes for which this species is well known were all readily apparent.

It was the consistent and reproducible comprehensiveness of the rapidly acquired small molecule fingerprint in each of the biological samples surveyed in this work that was exploited to conduct successful species classification using multivariate statistical analysis tools. Using supervised methods such as PCA, KDA and KPCA, we determined that a small number of principal components could be used to account for ~40% - 90% of the observed variance, with LOOCVs from 80 - 100% probability depending on the sample analyzed. Hierarchical cluster analysis was applied to the entire mass spectral data set in each case as an unbiased approach to assess the extent to which the DART-MS-derived chemical fingerprint could enable species classification. The resulting dendrograms showed that in all cases, striking species level separations were accomplished, demonstrating that genomic distinctions between even closely related species manifest themselves in small molecule profile differences. A number of previous reports have demonstrated that hierarchical clustering of the type used here can be exploited for classification purposes. However, in the majority of these cases, distinguishing biomarkers or specific spectral features, rather than the entire small molecule fingerprint, were used. A recurring observation in these studies was the appearance of misclassifications, a not too unexpected consequence of the fact that (a) the selected principal components accounted for less than 100% of the variance; and (b) the information content of the chemical data that was used as the input for multivariate statistical analysis processing was not comprehensive, in that it was acquired using extracts, or the analysis was performed by a method in which certain molecules were preferentially detected over others. For the samples used in this work, no misclassifications were observed when the entire mass spectral dataset was used. This suggests that small molecule fingerprint-based classifications that can reflect genome differences are best acquired using the full fingerprint, rather than a subset of salient features. Of note is the fact that this method does not require that the identities of the fingerprint components be known. Nevertheless, the knowledge of the molecular weights and formulas of distinguishing molecules provides important information that can be used to eventually determine compound identity.

In summary, we have devised a rapid high throughput method for species identification and classification based on chemometric analysis of comprehensive DART-TOF-MS derived chemical signatures. The method entails introduction of the sample to the open air space between the DART ion source and the mass spectrometer inlet, followed by chemometric processing using the entire mass spectral dataset. A range of both polar and non-polar chemotypes are instantaneously detected, and matter in various forms (i.e. solid, liquid or gaseous) is easily analyzed with no need to change the method of sample introduction. The comprehensive small molecule signatures obtained serve as the input for unsupervised hierarchical cluster processing software, a number of open source versions of which are freely and readily available. The result of this processing tool is identification and species level classification based on the entire DART-derived chemical fingerprint. This methodology circumvents some of the pitfalls of the data selection bias that can accompany the use of supervised methods of statistical analysis on the one hand, and the deficiencies introduced by other instrument/chemical methods (such as extraction) on the other. Furthermore, it is significantly faster than conventional methods and can yield results from start to finish (including statistical analysis), in less than 3 min per sample. Given that the type of genome classification results consistently observed here are most often acquired using gene sequence information, and the time and resources required to generate it, the method outlined here provides a significant advancement in the determination of species level classifications. It supplies further evidence that inherent in the metabolome is the information content required to determine species level distinctions. In this work, we show the application of this methodology for rapid species-level identification of: endangered woods; biofuel feedstocks; insect puparial cases; plants; and tree species. These applications fall within the fields of forensic science, agronomy, agriculture, natural products chemistry, plant biochemistry and fuel chemistry among others, and it is anticipated that it could easily be used to further discoveries in a myriad of other areas.

## Methods

### Instrumentation

An *AccuTOF* (JEOL Ltd., Akishima Japan) time-of-flight mass spectrometer equipped with a Direct Analysis in Real Time (DART) ion source (Ionsense LLC, Saugus, MA) was used for all measurements. Mass spectra were stored by the JEOL *Mass Center* data acquisition software at a rate of 1 per second for the *m/z* range 60 to 1000. The mass spectrometer resolving power was 6000 (FWHM) for protonated reserpine at *m/z* 609.2812. The atmospheric pressure interface (API) conditions for positive-ion measurements were: orifice 1 = 20 V, ring lens = orifice 2 = 5 V. The RF ion guide voltage (“Peaks Voltage”) was set to 600 V to permit analysis of ions greater than approximately *m/z* 60. For all analyses except those of the seeds and Eucalyptus leaves, a sample of poly(propylene glycol) with average molecular weight of 600, also referred to as “polyethylene glycol” (PEG 600), was measured in each data file as a reference standard for mass calibration. For the remaining samples, Jeffamine M600 (Huntsman, The Woodlands, TX) was used as the calibrant. Unless otherwise stated, the DART was operated with helium and a gas heater setting of 350 °C. Sample extracts were analyzed by exposing the closed end of a Corning Pyrex melting point capillary tube (Capitol Scientific, Austin TX USA) that had been dipped into the extract, to the open air space between the ion source and the mass spectrometer inlet.

### Mass spectral data processing

Data processing operations, including mass calibration, centroiding, spectral averaging and background subtraction were carried out with *TSSPro3* software (Shrader Software Solutions). *Mass Mountaineer* software (RBC Software, Portsmouth, NH) was used for classification chemometrics including heat maps, principal component analysis (PCA) and linear and kernel discriminant analysis (LDA and KDA respectively). Heat maps exported from *Mass Mountaineer* were imported into *Cluster 3.0* and *Java Treeview* (Stanford University) for hierarchical clustering analysis.

### Sample preparation and sample analysis

#### Dalbergia species

Because of the common practice of using a single name to refer to multiple species within the *Dalbergia* genus, and the fact that many of the samples we analyzed are rare and illegal to trade, we conducted our analyses on samples from xylarium collections whose species identities had been verified. We then compared these to samples from commercial sources. Wood samples of known identity were sourced from the USDA Forest Product Laboratory (FPL), the USDA Animal and Plant Health Inspection Service (APHIS), the Oregon State University Xylarium (OSU), La Xiloteca del Instituto de Biología, UNAM, Mexico City, México (XIB), Eisenbrand Inc. Exotic Hardwoods, Torrance, CA, USA (EIEH), Cook Woods, Klamath Falls, OR, USA (CW), Carlton McLendon Inc., Atlanta, GA (CMI), PFC Shanty Navarro Hurtado, the Brazilian Federal Police (SNH), and the Botany collection at the University of South Carolina (USC). Furthermore, samples from multiple countries (Mexico, Guatemala, Nicaragua, Panama, Costa Rica and Brazil) were analyzed. The number of replicates that could be analyzed depended upon and was limited by species availability. The comprehensive list appears in Supplementary Table 1. Briefly, 11 *D. granadillo*, 34 *D. retusa*, 22 *P. yucatanum,* 21 *C. echinata* and 12 *C. playloba* species were analyzed. For sampling, wood slivers were shaved from the heartwood of the reference specimens and placed directly in the DART helium gas stream for six seconds each. A mass calibration standard of polyethylene glycol 600 (Ultra, Kingstown RI) was run between every 5^th^ sample. For each species, sampling was conducted in replicates of 8-9.

#### Biofuel feedstocks

Soy-derived, canola-derived, and mixed feedstock biodiesels were obtained from Minnesota Soybean Processors (Brewster MN, USA), Archer Daniels Midland (Decatur IL, USA), and Future Fuel (Batesville, AR, USA), respectively. Non-commercial biodiesel samples were supplied by the United States Department of Agriculture, National Center for Agricultural Utilization Research, Agricultural Research Service (Peoria IL, USA). Hexane used to dilute samples for analysis was purchased from VWR (Denver CO, USA) and used as received. Biodiesel samples were measured by dipping the closed end of a melting point capillary tube into hexane solutions of each feedstock (30 μL of feedstock dissolved in 100 μL of hexane), and suspending the tube between the mass spectrometer inlet and the ion source. Solutions were sampled by DART-TOF-MS as described above in replicates of 5 for each feedstock.

#### Puparial cases

Puparial cases were provided by Dr. Jeffery Tomberlin (Texas A&M University, College Station TX USA) and Dr. Eric Benbow (Michigan State University, USA). Individual insect cases were deposited into vials containing 300  μL of hexane (Thermo Fisher Scientific, Waltham MA USA) and allowed to stand for 5 min before DART sampling of the extract using the sealed end of a melting point capillary. The DART exit grid potential was set to +250 V. For every species, 5 cases were sampled in replicates of 5 each.

#### Datura, Brugmansia and Hyocyamus species differentiation

*B. arborea* and *D. ferox* seeds were purchased from Georgia Vines (Claxton GA, USA). *H. niger*, *D. stramonium*, and *D. inoxia* seeds were purchased from Horizon Herbs (Williams OR, USA). Individual seeds were sampled by DART-TOF-MS using a vacuum tweezer apparatus to suspend the seeds between the ion source and the mass spectrometer inlet. For analysis, seeds were cut in half and one open half of the seed was oriented so that if faced the DART ion source. For each species, mass spectra were measured in replicates of 5.

#### Eucalypt analysis

The species of Eucalyptus analyzed were *Eucalyptus polyanthemos* (10 plants with 5 replicates from each plant), *E. bridgesiana* apple (2 plants with 25 replicates from each), *E. globulus* (10 plants with 5 replicates each), *E. citriodora* (10 plants with 5 replicates each), and *E. cineraria* (4 plants with 17 replicates each). All plants except *E. bridesiana* were purchased from Companion Plants Inc. (Athens, OH, USA). *E. bridgesiana* was purchased from Faddegon’s Nursery (Latham, NY, USA). Plant leaves were sampled by removal of 6 mm diameter circular segments from the leaves of live soil bound plants with a paper hole punch and suspending the leaf sample in the open air space between the ion source and the mass spectrometer inlet.

### Multivariate statistical analysis

Mass-calibrated and centroided mass spectra were exported from the data processing software (TSSPro3, Shrader Software Solutions, Detroit, MI) as text files for entry into the elemental composition and classification software (Mass Mountaineer, RBC Software, Portsmouth, NH, available from mass-spec-software.com). Principal components were calculated by using the correlation matrix. Abundances used for classification were selected from each mass spectrum for the indicated number of peaks having *m/z* values within 0.005–015 u of the target *m/z* value. Heat maps were rendered as text files for import into Cluster 3.0 for single linkage hierarchical cluster analysis (Michiel de Hoon, University of Tokyo, adapted from the Cluster Program written by Michael Eisen, Stanford University, available at http://bonsai.hgc.jp/~mdehoon/software/cluster/software.htm). Dendrograms were observed using Java Treeview (written by Alok Saldanha, available at http://jtreeview.sourceforge.net/).

## Additional Information

**How to cite this article**: Musah, R. A. *et al.* A High Throughput Ambient Mass Spectrometric Approach to Species Identification and Classification from Chemical Fingerprint Signatures. *Sci. Rep.* **5**, 11520; doi: 10.1038/srep11520 (2015).

## Supplementary Material

Supplementary Information
