# Supplementary material for: A High Throughput Ambient Mass Spectrometric Approach to Species Identification and Classification from Chemical Fingerprint Signatures
Source: Sci Rep. 2015 Jul 9;5:11520. doi: 10.1038/srep11520 (PMC4648398; doi:10.1038/srep11520)
Supplement: Supplementary Information [file srep11520-s1.pdf]

A High Throughput Ambient Mass Spectrometric Approach to Species Identification and Classification from Chemical Fingerprint Signatures

SUPPLEMENTARY INFORMATION

Authors:

Rabi A. Musah[1]*, Edgard O. Espinoza[2], Robert B. Cody[3], Ashton D. Lesiak[1], Earl D. Christensen[4], Hannah E. Moore[5], Simin Maleknia[6] and Falko P. Drijfhout[5]

Affiliations:

[1]Department of Chemistry, University at Albany, State University of New York, 1400 Washington Avenue, Albany, NY 12222 USA

[2]U.S. National Fish and Wildlife Forensics Laboratory, 1490 East Main Street, Ashland, OR, 97520-1310, USA

[3]JEOL USA Inc., 11 Dearborn Road, Peabody, MA 01960 USA

[4]National Renewable Energy Laboratory, 15013 Denver West Parkway, MS-1634, Golden, CO 80401 USA

[5]Department of Chemical Ecology, School of Physical and Geographical Science, Keele University, Keele ST5 5BG, UK

[6]School of Biological, Earth & Environmental Sciences, University of New South Wales, Sydney, Australia

[7]Department of Chemistry, Keele University, United Kingdom ST5 5BG

SUPPLEMENTARY INFORMATION

Supplementary Figure 1. Positive ion mode DART-TOF-MS of *Dalbergia* species. Detailed mass information including *m/z* values, their corresponding abundances and tentative structural assignments is presented in Supplementary Tables 2a-e. Each spectrum is representative of the species tested. Sample size for each species is given in Supplementary Table 1. The *D. granadillo* samples were authenticated specimens.

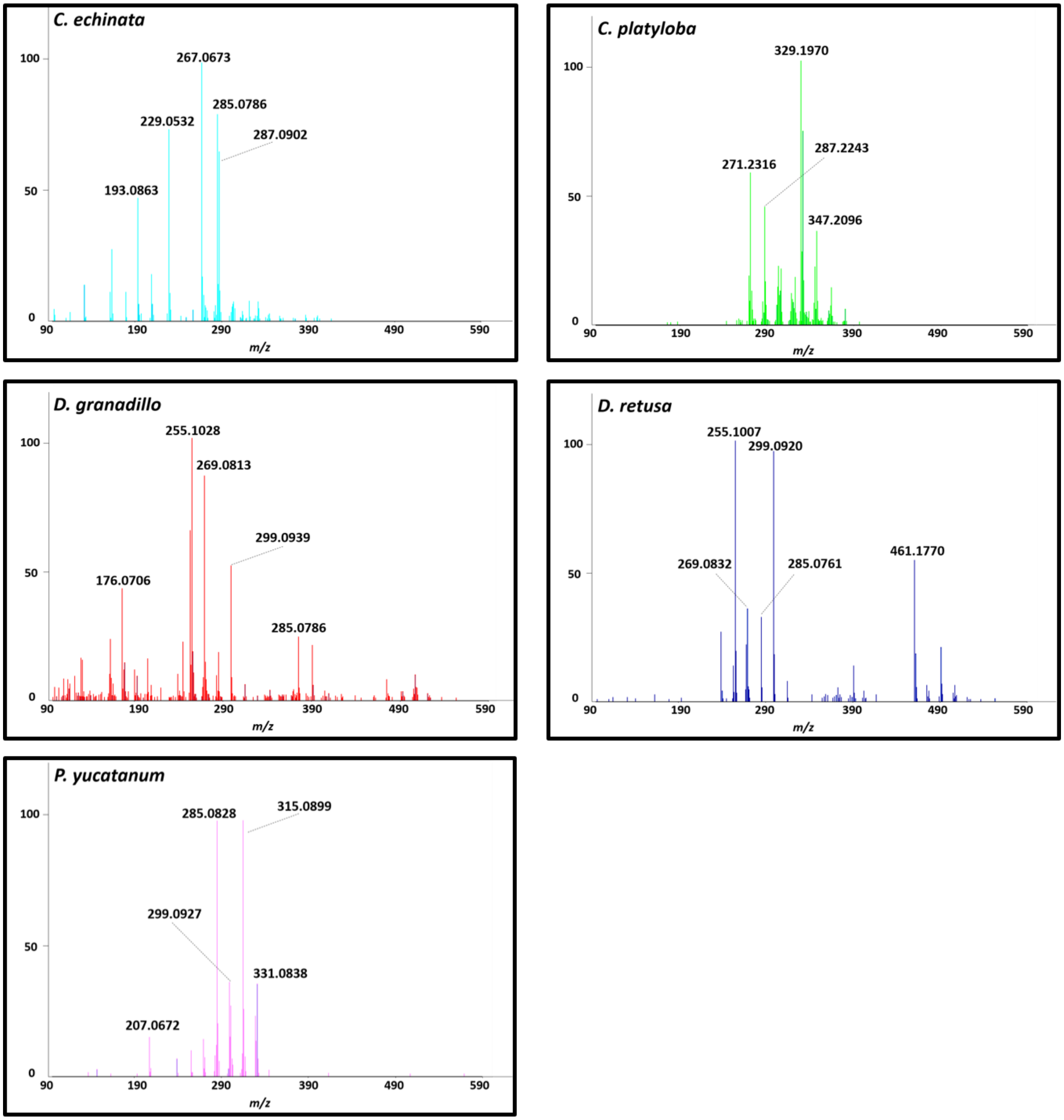

Supplementary Figure 2. Positive ion mode DART-TOF-MS of the indicated biofuel feedstocks dissolved in hexane. Detailed mass information including *m/z* values, their corresponding abundances and tentative structural assignments is presented in Supplementary Table 3. Each spectrum represents a single measurement.

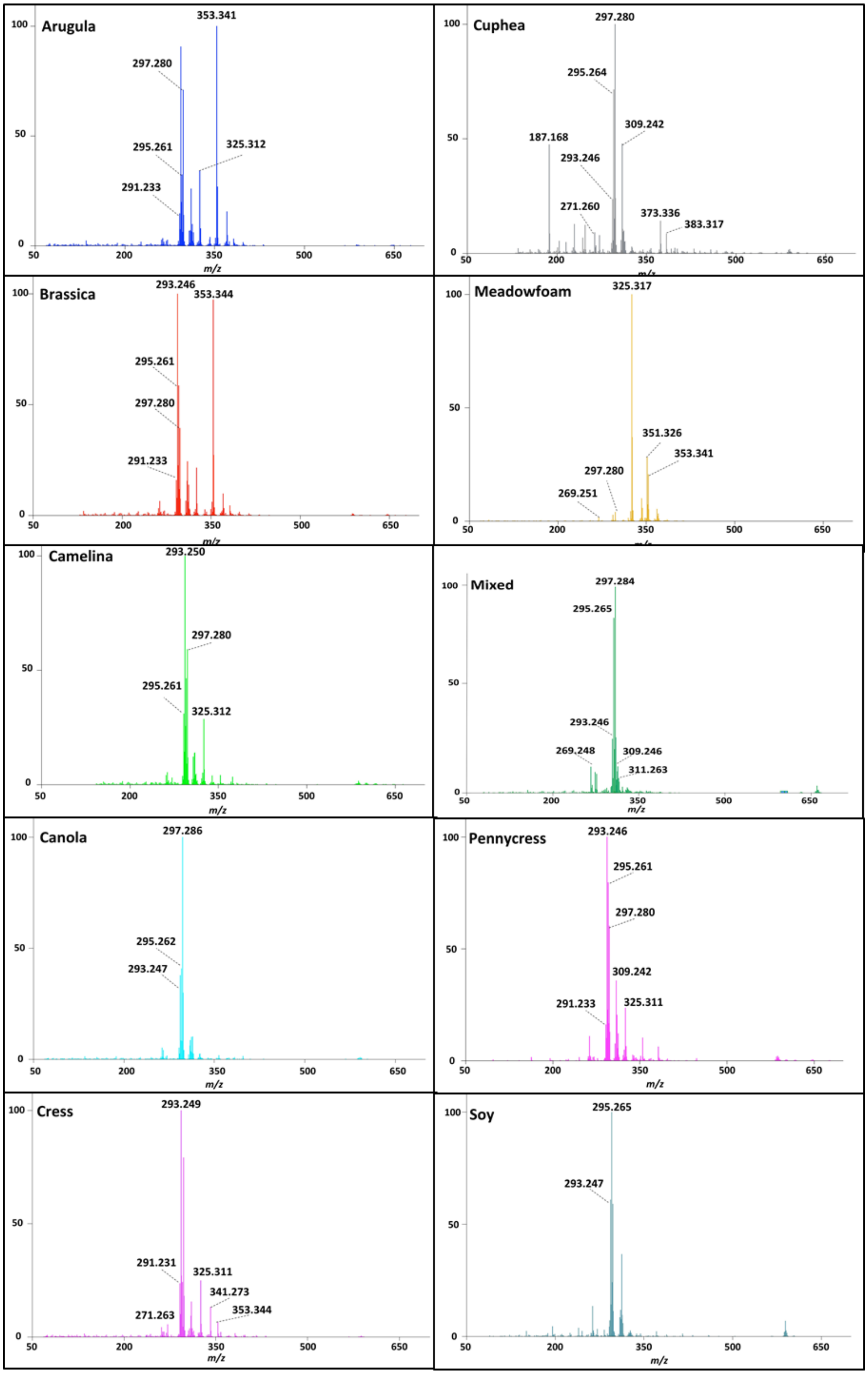

Supplementary Figure 3. DART-TOF mass spectra in negative ion mode of hexane extracts of puparial cases of the indicated flies. Detailed mass information including *m/z* values, their corresponding abundances and tentative structural assignments is presented in Supplementary Table 4. Each spectrum represents an average of five individual analyses.

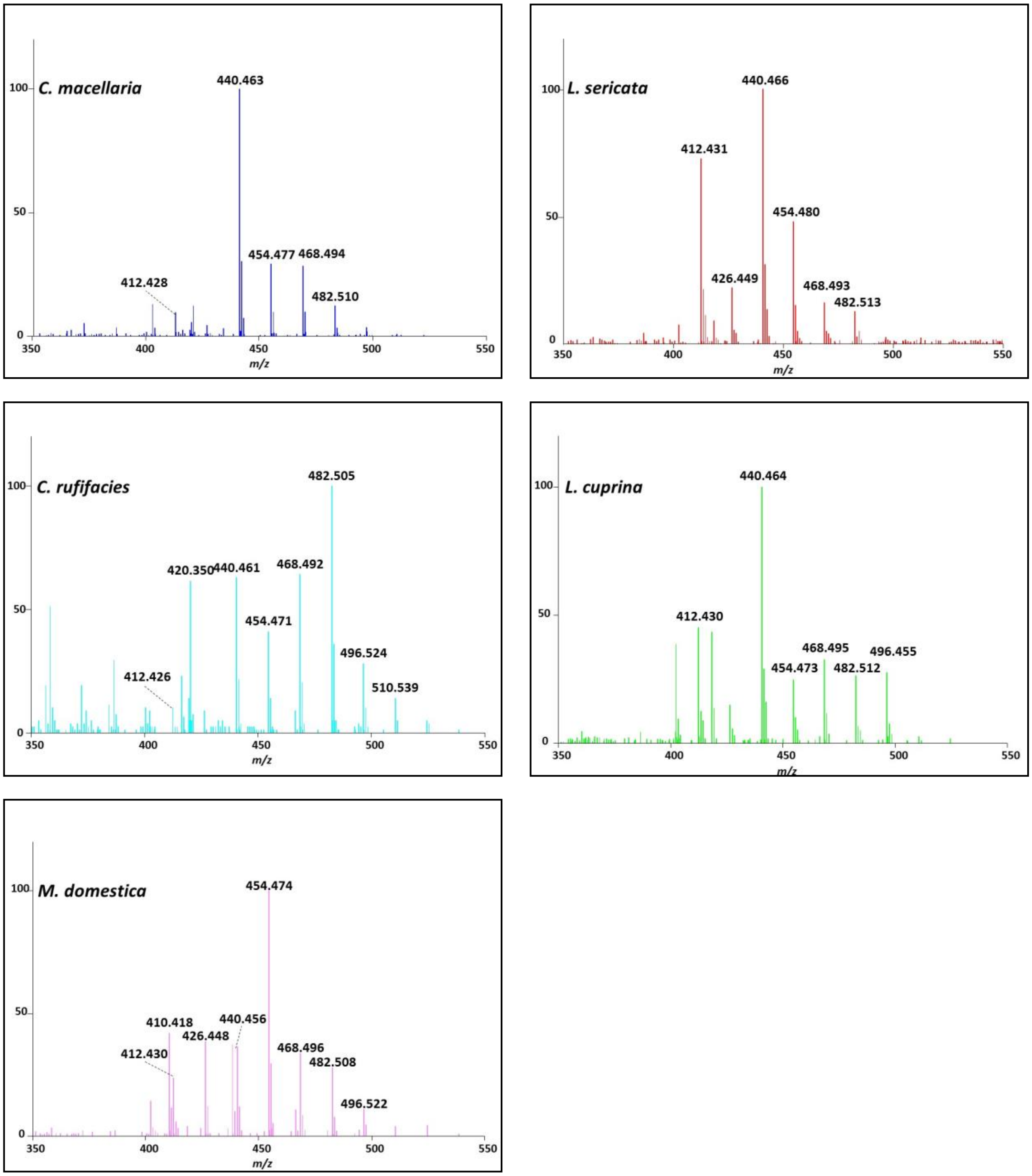

Supplementary Figure 4. Mass spectrum of the hexane extract of the Sample B puparial casing unknown. This sample exhibited a strong cholesterol $[M+O_2]^-$ peak (i.e. *m/z* 418.351). Cluster analysis correctly classified it as being derived from *C. rufifacies* despite the cholesterol interference.

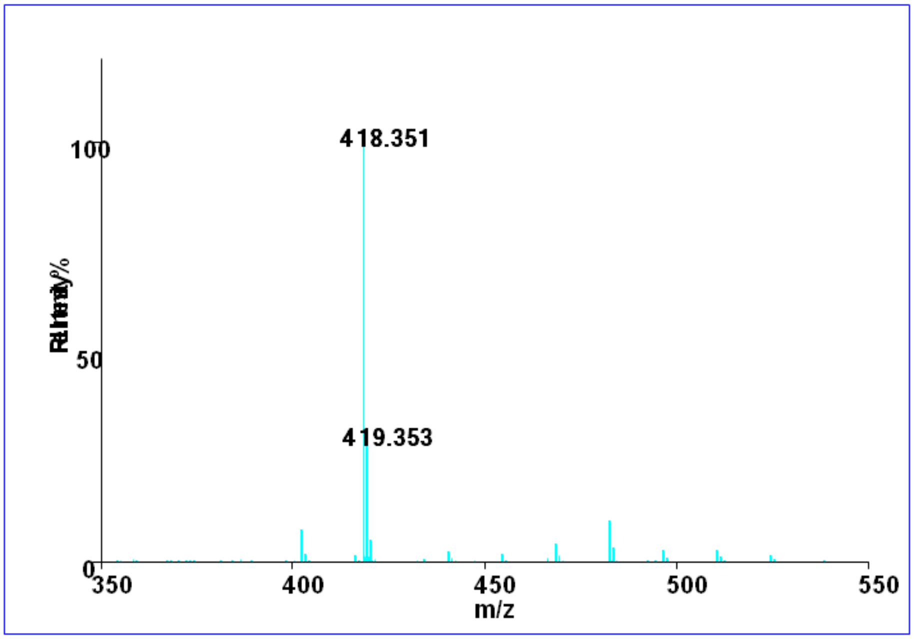

Supplementary Figure 5. Positive ion mode DART-TOF-MS spectra of the indicated *Datura*, *Brugmansia* and *Hyocyamus* species. Each spectrum represents an average of five individual analyses. The molecular formulas corresponding to the indicated high resolution masses are presented in Supplementary Tables 5a-5e.

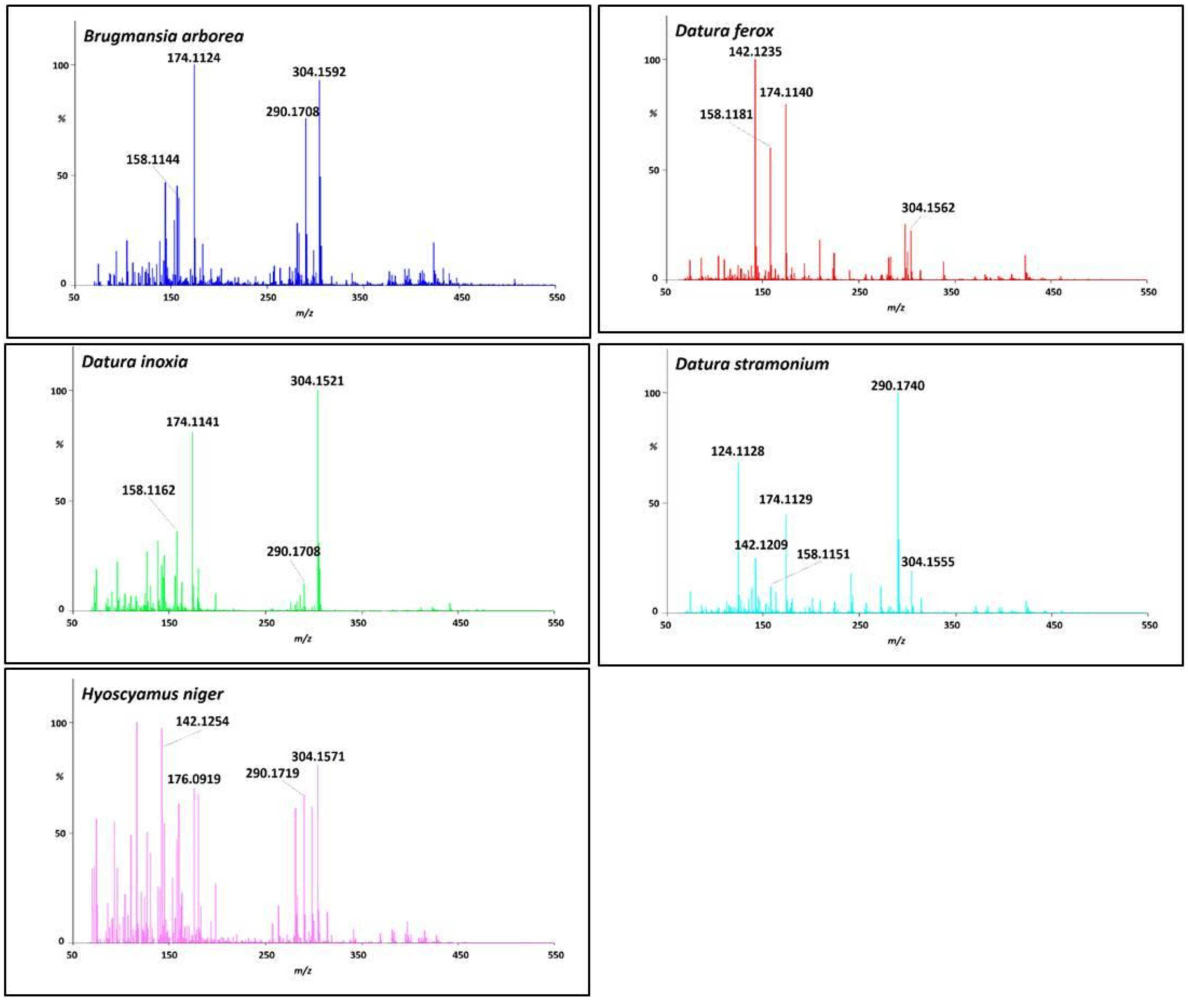

Supplementary Figure 6. **.** Positive ion mode DART-TOF-MS spectra of leaves of the indicated Eucalypt species. Each spectrum represents an average of five individual analyses. The molecular formulas corresponding to the indicated high resolution masses are presented in Supplementary Tables 6a-6e.

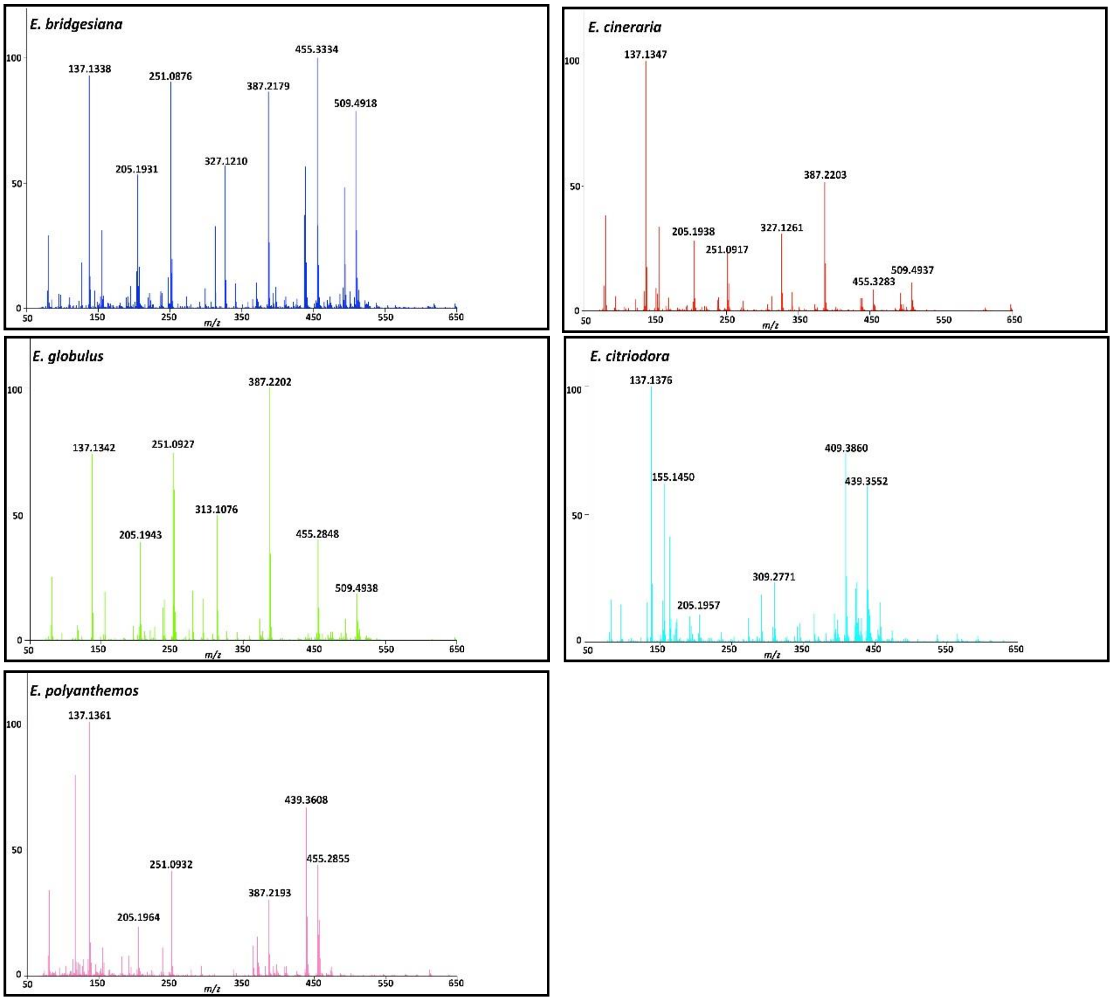

SUPPLEMENTARY TABLES

Supplementary Table 1. *Dalbergia* species analyzed in this study that have been described by the common name "granadillo."

**Geographic Provenance**	**Species**	**CITES**	**n**	Country of Origin	Source[a] (n)
S. America	*Caesalpinia echinata*	App II	18	Brazil<br>1 Unknown	EIEH (1)<br>FPL (4)<br>CMI (10)<br>APHIS (1)<br>USC (1)<br>SNH (1)
C. America	*Dalbergia retusa*	App II	34	20 Mexico<br>8 Panama<br>2 Nicaragua<br>1 Costa Rica<br>2 C America<br>1 unknown	OSU (1)<br>FPL (13)<br>XIB (1)<br>EIEH (19)
C. America	*Dalbergia granadillo*	App II	11	8 Mexico<br>1 Guatemala<br>1 Nicaragua<br>1 unknown	FPL (5)<br>XIB (5)<br>CW (1)
C. America	*Caesalpinia platyloba*		12	Mexico	XIB (2)<br>EIEH (10)
C. America	*Platymiscium yucatanum*		20	Mexico	XIB (6)<br>CMI (14)

[a]Source of the specimens included the USDA Forest Product Laboratory (FPL), USDA Animal and Plant Health Inspection Service (APHIS), Oregon State University Xylarium (OSU), La Xiloteca del Instituto de Biología, UNAM, Mexico City, México (XIB), Eisenbrand Inc. Exotic Hardwoods, Torrance, CA, USA (EIEH) Cook Woods, Klamath Falls, OR, USA (CW), Carlton McLendon Inc., Atlanta, GA (CMI), PFC Shanty Navarro Hurtado, Brazilian Federal Police (SNH), and the Botany collection at the University of South Carolina (USC). Commercially obtained specimens were verified by either anatomical analysis or statistical consensus results with known curated specimens.

Supplementary Tables 2a-c. Mass spectral data associated with the positive ion mode mass spectra of *Dalbergia* species woods shown in Supplementary Figure 1.

Supplementary Table 2a. DART-TOF-MS data for selected peaks in the spectrum of *D. retusa* shown in Supplementary Figure 1.

Formula[a]	Adduct	Measured	Calculated	Difference[b]	Abundance
$C_{16}H_{14}O_3$[c]	$+H^+$	255.1007	255.1021	-1.4	100.0
$C_{16}H_{12}O_4$[d]	$+H^+$	269.0832	269.0814	1.8	35.6
$C_{16}H_{12}O_5$[e]	$+H^+$	285.0761	285.0763	-0.2	32.4
$C_{17}H_{14}O_5$[f]	$+H^+$	299.0921	299.0920	-0.1	96.1
$C_{17}H_{14}O_6$[g]	$+H^+$	315.0888	315.0869	1.9	7.9

[a]Compound formulas were consistent with those of the indicated molecules (cited below) that were previously isolated from *Dalbergia* species[13,14]. [b]Measured masses fell within 5 mmu of the calculated masses. [c]Compound formula is consistent with previously identified compounds found in *Dalbergia* species, including 4-methoxydalbergione, parvifuran, dalbergichromene, obtusaquinone, 4-hydroxy2-methoxychalcone, and isoparvifuran. [d]Compound formula is consistent with previously identified compounds found in *Dalbergia* species, including formoneetin, isodalbergin, dalbergin, and 7-hydroxy-6-methoxyflavone. [e]Compound formula is consistent with previously identified compounds found in *Dalbergia* species, including biochanin A, prunetin, melanin, calycosin, 3-methoxydaidzein, glycitein, retusin, and maackiain. [f]Compound formula is consistent with previously identified compounds found in *Dalbergia* species, including afrormosin, 8-O-methylretusin. [g]Compound formula is consistent with previously identified compounds found in *Dalbergia* species, including, odoratin, 7-O-methyltectorigenin, 4-hydroxypterocarpin, onogenin, and 4-methoxymaackiain.

**Supplementary Table 2b.** DART-TOF-MS data for selected peaks in the spectrum of *D. granadillo* shown in Supplementary Figure 1.					
**Formula**[a]	**Adduct**	**Measured**	**Calculated**	**Difference**[b]	**Abundance**
$C_{10}H_9NO_2$[c]	$+H^+$	176.0706	176.07115	-0.6	42.9
$C_{11}H_{11}NO_2$[d]	$+H^+$	190.0879	190.0868	1.1	11.8
$C_{14}H_{12}O_4$[e]	$+H^+$	145.0826	245.0814	1.2	22.5
$C_{16}H_{14}O_3$[f]	$+H^+$	255.1007	255.1021	-1.4	100.0
$C_{16}H_{12}O_4$[g]	$+H^+$	269.0832	269.0814	1.8	35.6
$C_{16}H_{12}O_5$[h]	$+H^+$	285.0761	285.0763	-0.2	32.4
$C_{17}H_{14}O_5$[i]	$+H^+$	299.0921	299.0920	-0.1	96.1
$H_{19}H_{18}O_8$[j]	$+H^+$	375.1123	375.1080	4.3	24.3

[a]Compound formulas were consistent with those of the indicated molecules (cited below) that were previously isolated from *Dalbergia* species[13,14]. [b]Measured masses fell within 5 mmu of the calculated masses. [c]Compound formula is consistent with indole-3-acetic acid, previously identified in *Dalbergia* species. [d]Compound formula is consistent with methyl indole-3-acetate, previously identified in *Dalbergia* species. [e]Compound formula is consistent with cearoin, previously identified in *Dalbergia* species. [f]Compound formula is consistent with previously identified compounds found in *Dalbergia* species, including 4-methoxydalbergione, parvifuran, dalbergichromene, obtusaquinone, 4-hydroxy2-methoxychalcone, and isoparvifuran. [g]Compound formula is consistent with previously identified compounds found in *Dalbergia* species, including formoneetin, isodalbergin, dalbergin, and 7-hydroxy-6-methoxyflavone. [h]Compound formula is consistent with previously identified compounds found in *Dalbergia* species, including biochanin A, prunetin, melanin, calycosin, 3-methoxydaidzein, glycitein, retusin, and maackiain. [i]Compound formula is consistent with previously identified compounds found in *Dalbergia* species, including afrormosin, 8-O-methylretusin. [j]Compound formula is consistent with previously identified compounds found in *Dalbergia* species, including, isocaviunin, and caviunin.

**Supplementary Table 2c**. DART-TOF-MS data for observed peaks in the spectra of the *Dalbergia* species woods *C. platyloba*, *C. echinata* and *P. yucatanum* shown in Supplementary Figure 1.		
C. platyloba	*C. echinata*	*P. yucatanum*
279.232	279.232	285.083
287.224	287.224	299.093
329.197	329.197	315.090
331.213	331.213	331.084
347.210	347.210	
364.234	364.234	

**Supplementary Table 3.** Mass spectral data associated with the positive ion mode DART-TOF-MS spectra of hexane extracts of the biodiesel feedstocks shown in Supplementary Figure 2. Unless otherwise indicated, all ions were observed as protonated adducts of fatty acid methyl esters (FAMEs).					
**Biodiesel feedstock**	**Formula**	**Measured**	**Calculated**	**Difference**	**Relative Abundance**
Arugula	$C_{19}H_{31}O_2$ (C18:4)	291.2327	291.2324	0.3	14.6
	$C_{19}H_{31}O_2$ (C18:4)	293.2464	293.2481	-1.7	90.8
	$C_{19}H_{35}O_2$ (C18:2)	295.2610	295.2637	-2.7	32.5
	$C_{19}H_{37}O_2$ (C18:1)	297.2803	297.2794	0.9	70.9
	$C_{21}H_{41}O_2$ (C20:1)	325.3118	325.3107	1.1	34.2
	$C_{23}H_{45}O_2$ (C22:1)	353.3415	353.3420	-0.5	100.0
Brassica	$C_{19}H_{31}O_2$ (C18:4)	291.2327	291.2324	0.3	15.9
	$C_{19}H_{33}O_2$ (C18:3)	293.2465	293.2481	-1.6	100.0
	$C_{19}H_{35}O_2$ (C18:2)	295.2612	295.2637	-2.5	58.6
	$C_{19}H_{37}O_2$ (C18:1)	297.2801	297.2794	0.7	39.5
	$C_{23}H_{45}O_2$ (C22:1)	353.3441	353.3420	2.1	97.4
Camelina	$C_{19}H_{33}O_2$ (C18:3)	293.2497	293.2481	1.6	100.0
	$C_{19}H_{35}O_2$ (C18:2)	295.2609	295.2637	-2.8	46.4
	$C_{19}H_{37}O_2$ (C18:1)	297.2801	297.2794	0.7	59.1
	$C_{21}H_{41}O_2$ (C20:1)	325.3117	325.3107	1.0	28.7
Canola	$C_{19}H_{33}O_2$ (C18:3)	293.2473	293.2481	-0.8	37.8
	$C_{19}H_{35}O_2$ (C18:2)	295.2622	295.2637	-1.5	40.8
	$C_{19}H_{37}O_2$ (C18:1)	297.2828	297.2794	3.4	100.0
Cress	$C_{17}H_{35}O_2$ (C16:0)	271.2630	271.2637	-0.7	5.5
	$C_{19}H_{31}O_2$ (C18:4)	291.2311	291.2324	-1.3	23.7
	$C_{19}H_{33}O_2$ (C18:3)	293.2488	293.2481	0.7	100.0
	$C_{21}H_{41}O_2$ (C20:1)	325.3106	325.3107	-0.1	25.0
	$C_{23}H_{45}O_2$ (C22:1)	353.3435	353.3420	1.5	6.5
Cuphea	$C_{11}H_{23}O_2$ (C10:0)	187.1684	187.1698	-1.4	47.4
	$C_{17}H_{35}O_2$ (C16:0)	271.2603	271.2637	-3.4	8.0
	$C_{19}H_{33}O_2$ (C18:3)	293.2455	293.2481	-2.6	23.7
	$C_{19}H_{35}O_2$ (C18:2)	295.2638	295.2637	0.1	71.5
	$C_{19}H_{37}O_2$ (C18:1)	297.2799	297.2794	0.5	100.0
	$C_{19}H_{33}O_3$[b]	309.2419	309.2430	-1.1	48.0
Meadowfoam	$C_{17}H_{33}O_2$ (C16:1)	269.2510	269.2481	2.9	1.6
	$C_{19}H_{37}O_2$ (C18:1)	297.2805	297.2794	1.1	4.0
	$C_{21}H_{41}O_2$ (C20:1)	325.3168	325.3107	6.1	100.0
	$C_{23}H_{43}O_2$ (C22:2)	351.3256	351.3263	-0.7	28.2
	$C_{23}H_{45}O_2$ (C22:1)	353.3413	353.3420	-0.7	20.7
Mixed	$C_{17}H_{33}O_2$ (C16:1)	269.2476	269.2481	-0.5	10.0
	$C_{19}H_{33}O_2$ (C18:3)	293.2464	293.2481	-1.7	26.3

	$C_{19}H_{35}O_2$ (C18:2)	295.2651	295.2637	1.4	84.9
	$C_{19}H_{37}O_2$ (C18:1)	297.2843	297.2794	4.9	100.0
	$C_{19}H_{33}O_3$[b]	309.2465	309.2430	3.5	14.8
	$C_{19}H_{35}O_3$[b]	311.2627	311.2586	4.1	10.4
Pennycress	$C_{19}H_{31}O_2$ (C18:4)	291.2326	291.2324	0.2	16.1
	$C_{19}H_{33}O_2$ (C18:3)	293.2462	293.2481	-1.9	100.0
	$C_{19}H_{35}O_2$ (C18:2)	295.2611	295.2637	-2.6	79.7
	$C_{19}H_{37}O_2$ (C18:1)	297.2800	297.2794	0.6	60.3
	$C_{19}H_{33}O_3$[b]	309.2421	309.2430	-0.9	35.8
	$C_{21}H_{41}O_2$ (C20:1)	325.3113	325.3107	0.6	23.6
Soy	$C_{19}H_{33}O_2$ (C18:3)	293.2466	293.2481	-1.5	60.9
	$C_{19}H_{35}O_2$ (C18:2)	295.2651	295.2637	1.4	100.0
	$C_{19}H_{37}O_2$ (C18:1)	297.2805	297.2794	1.1	59.1

[a]The formula is consistent with this being a monoglyceride. [b]The formula is consistent with this being an oxo- or hydroxy-fatty acid methyl ester.

Supplementary Table 4. Mass spectral data associated with the negative ion mode DART-TOF-MS spectra of hexane extracts of the insect puparial casings shown in Supplementary Figure 3. All ionized hydrocarbons were observed as O_2^- adducts.

Blowfly species	Formula	Measured	Calculated	Difference	Abundance
Chrysomya rufifacies	$[C_{27}H_{56} + O_2]^-$	412.426	412.429	-3	10.3
	$[C_{29}H_{60} + O_2]^-$	440.461	440.460	1	62.8
	$[C_{30}H_{62} + O_2]^-$	454.471	454.475	-4	41.0
	$[C_{31}H_{64} + O_2]^-$	468.492	468.491	1	64.1
	$[C_{32}H_{66} + O_2]^-$	482.505	482.506	-1	100.0
	$[C_{33}H_{68} + O_2]^-$	496.524	496.522	2	28.2
	$[C_{34}H_{70} + O_2]^-$	510.539	510.538	1	14.1
Lucilia sericata	$[C_{27}H_{56} + O_2]^-$	412.431	412.429	2	68.0
	$[C_{28}H_{58} + O_2]^-$	426.449	426.444	5	14.9
	$[C_{29}H_{60} + O_2]^-$	440.466	440.460	6	100.0
	$[C_{30}H_{62} + O_2]^-$	454.480	454.475	5	78.9
	$[C_{31}H_{64} + O_2]^-$	468.493	468.491	2	35.1
	$[C_{32}H_{66} + O_2]^-$	482.513	482.506	7	31.4
L. cuprina	$[C_{27}H_{56} + O_2]^-$	412.430	412.429	1	54.9
	$[C_{29}H_{60} + O_2]^-$	440.464	440.460	4	100.0
	$[C_{30}H_{62} + O_2]^-$	454.473	454.475	-2	19.9
	$[C_{31}H_{64} + O_2]^-$	468.495	468.491	4	21.0
	$[C_{32}H_{66} + O_2]^-$	482.512	482.506	6	16.0
	$[C_{31}H_{60} + O_2]^-$	496.455	496.449	-6	14.2
Cochliomya macellaria	$[C_{27}H_{56} + O_2]^-$	412.428	412.429	-1	7.8
	$[C_{29}H_{60} + O_2]^-$	440.463	440.460	3	100.0
	$[C_{30}H_{62} + O_2]^-$	454.477	454.475	2	35.8
	$[C_{31}H_{64} + O_2]^-$	468.494	468.491	3	34.6
	$[C_{32}H_{66} + O_2]^-$	482.510	482.506	4	15.1
Musca domestica	$[C_{27}H_{54} + O_2]^-$	410.418	410.412	6	91.6
	$[C_{27}H_{56} + O_2]^-$	412.428	412.429	-1	32.7
	$[C_{28}H_{58} + O_2]^-$	426.448	426.444	4	70.6
	$[C_{29}H_{58} + O_2]^-$	438.444	438.444	0	54.7
	$[C_{29}H_{60} + O_2]^-$	440.456	440.460	-4	51.5
	$[C_{30}H_{62} + O_2]^-$	454.474	454.475	-1	100.0
	$[C_{31}H_{64} + O_2]^-$	468.496	468.491	5	32.8
	$[C_{32}H_{66} + O_2]^-$	482.508	482.506	2	27.8
	$[C_{33}H_{68} + O_2]^-$	496.522	496.522	0	12.2

Supplementary Tables 5a – 5e. Mass spectral data associated with the positive ion DART-TOF-MS spectra of *D. stramonium*, *D. inoxia*, *D. ferox*, *B. arborea* and *H. niger* that are shown in Supplementary Figure 5.

Supplementary Table 5a. DART-TOF-MS data for selected peaks in the spectrum of *B. arborea* shown in Supplementary Figure 5.

Formula	Adduct	Measured	Calculated	Difference[a]	Abundance
$C_8H_{15}NO$[b]	$+H^+$	142.1276	142.1232	-4.4	11.4
$C_8H_{13}NO_2$[c]	$+H^+$	156.1024	156.1025	0.1	45.2
$C_8H_{15}NO_2$[d]	$+H^+$	158.1144	158.1181	3.7	39.8
$C_6H_{12}O_6$[e]	$+NH_4^+$	198.1021	198.0978	-4.3	4.2
$C_{16}H_{19}NO_2$[f]	$+H^+$	258.1471	258.1494	2.3	1.1
$C_{13}H_{21}NO_5$[g]	$+H^+$	272.1479	272.1498	2.9	3.0
$C_{16}H_{21}NO_3$[h]	$+H^+$	276.1555	276.1600	4.5	6.5
$C_{16}H_{21}NO_2$[i]	$+NH_4^+$	277.1886	277.1916	3.0	2.1
$C_{18}H_{32}O_2$[j]	$+H^+$	281.2440	281.2481	4.1	28.3
$C_{17}H_{23}NO_3$[k]	$+H^+$	290.1708	290.1756	4.8	75.8
$C_{16}H_{19}NO_3$[l]	$+NH_4^+$	291.1749	291.1709	-4.0	23.3
$C_{18}H_{34}O_2$[m]	$+NH_4^+$	300.2898	300.2903	0.5	5.9
$C_{17}H_{12}NO_4$[n]	$+H^+$	304.1592	304.1549	-4.3	93.1

[a]Measured masses fell within 5 mmu of the calculated mass.

**Supplementary Table 5b.** DART-TOF-MS data for selected peaks in the spectrum of *D. ferox* shown in Supplementary Figure 5.					
**Formula**	**Adduct**	**Measured**	**Calculated**	**Difference[a]**	**Abundance**
$C_8H_{15}NO$[b]	$+H^+$	142.1235	142.1232	0.3	100.0
$C_8H_8O_3$[c]	$+H^+$	153.0558	153.0552	0.6	4.4
$C_8H_{13}NO_2$[d]	$+H^+$	156.1017	156.1025	-0.7	3.7
$C_8H_{15}NO_2$[e]	$+H^+$	158.1181	158.1181	-0.0	60.0
$C_6H_{12}O_6$[f]	$+NH_4^+$	198.0978	198.0978	1.2	2.3
$C_{13}H_{21}NO_2$[g]	$+H^+$	224.1656	224.1651	0.5	12.3
$C_{13}H_{21}NO_3$[h]	$+H^+$	240.1603	240.1600	0.3	4.4
$C_{13}H_{21}NO_4$[i]	$+H^+$	256.1521	256.1549	-2.8	1.2
$C_{18}H_{32}O_2$[j]	$+H^+$	281.2453	281.2481	-2.8	10.2
$C_{18}H_{34}O_2$[k]	$+H^+$	283.2613	283.2637	-2.4	10.6
$C_{17}H_{12}NO_4$[l]	$+H^+$	304.1577	304.1549	2.3	32.0

[a]Measured masses fell within 5 mmu of the calculated mass. [b]Compound formula is consistent with tropine, previously identified in *Datura* species[41]. [c]Compound formula is consistent with vanillin, previously identified in *Datura* species[41]. [d]Compound formula is consistent with scopoline, previously identified in *Datura* species[41]. [e]Compound formula is consistent with dihydroxytropane, previously identified in *Datura* species[41]. [f]Compound formula is consistent with hexose sugar, previously identified in *Datura* species[41]. [g]Compound formula is consistent with 3-tiglyloxytropane, previously identified in *Datura* species[41]. [h]Compound formula is consistent with 3-hydroxy-6-tigloyloxytropane, previously identified in *Datura* species[41]. [i]Compound formula is consistent with 3-tigloyloxy-6,7-dihydroxytropane, previously identified in *Datura* species[41]. [j]Compound formula is consistent with linoleic acid, previously identified in *Datura* species[42]. [k]Compound formula is consistent with oleic acid, previously identified in *Datura* species[42]. [l]Compound formula is consistent with scopolamine, previously identified in *Datura* species[41].

**Supplementary Table 5c.** DART-TOF-MS data for selected peaks in the spectrum of *D. inoxia* shown in Supplementary Figure 5.					
**Formula**	**Adduct**	**Measured**	**Calculated**	**Difference[a]**	**Abundance**
$C_8H_8O_3$[b]	$+H^+$	153.0591	153.0552	3.9	1.2
$C_8H_{13}NO_2$[c]	$+H^+$	156.1013	156.1025	-1.1	15.9
$C_8H_{15}NO_2$[d]	$+H^+$	158.1162	158.1181	-1.9	36.2
$C_6H_{12}O_6$[e]	$+NH_4^+$	198.0988	198.0978	1.0	7.7
$C_{16}H_{21}NO_3$[f]	$+H^+$	276.1586	276.1600	-1.4	4.1
$C_{17}H_{21}NO_3$[g]	$+H^+$	288.1571	288.1600	-2.9	1.9
$C_{17}H_{23}NO_3$[h]	$+H^+$	290.1708	290.1756	-4.8	12.4
$C_{16}H_{19}NO_3$[i]	$+NH_4^+$	291.1745	291.1709	3.6	2.3
$C_{18}H_{34}O_2$[j]	$+NH_4^+$	300.2917	300.2903	1.4	2.5
$C_{17}H_{12}NO_4$[k]	$+H^+$	304.1577	304.1549	2.8	100.0
$C_{17}H_{23}NO_4$[l]	$+H^+$	306.1674	306.1705	-3.1	19.2

[a]Measured masses fell within 5 mmu of the calculated mass. [b]Compound formula is consistent with vanillin, previously identified in *Datura* species[43]. [c]Compound formula is consistent with scopoline, previously identified in *Datura* species[41]. [d]Compound formula is consistent with dihydroxytropane, previously identified in *Datura* species[41]. [e]Compound formula is consistent with hexose sugar, previously identified in *Datura* species[41]. [f]Compound formula is consistent with norhyoscyamine, previously identified in *Datura* species[41]. [g]Compound formula is consistent with dihydroaposcopolamine, previously identified in *Datura* species[41]. [h]Compound formula is consistent with atropine, previously identified in *Datura* species[41]. [i]Compound formula is consistent with 3-acetoxy-6,7-epoxytropane, previously identified in *Datura* species[41]. [j]Compound formula is consistent with oleic acid, previously identified in *Datura* species[42]. [k]Compound formula is consistent with scopolamine, previously identified in *Datura* species[41]. [l]Compound formula is consistent with hydroxyhyoscyamine, previously identified in *Datura* species[41].

**Supplementary Table 5d.** DART-TOF-MS data for selected peaks in the spectrum of *D. stramonium* shown in Supplementary Figure 5.					
**Formula**	**Adduct**	**Measured**	**Calculated**	**Difference[a]**	**Abundance**
$C_8H_{15}NO$[b]	$+H^+$	142.1209	142.1232	-2.3	24.9
$C_8H_8O_3$[c]	$+H^+$	153.0580	153.0552	2.8	4.2
$C_8H_{13}NO_2$[d]	$+H^+$	156.0988	156.1025	-3.7	2.8
$C_8H_{15}NO_2$[e]	$+H^+$	158.1151	158.1181	-3.0	12.1
$C_6H_{12}O_6$[f]	$+NH_4^+$	198.0979	198.0978	0.1	2.6
$C_{13}H_{21}NO_2$[g]	$+H^+$	224.1649	224.1651	-0.2	5.2
$C_{17}H_{23}NO_3$[h]	$+H^+$	290.1740	290.1756	-1.6	100.0
$C_{18}H_{32}O_2$[i]	$+NH_4^+$	298.2730	298.2756	-1.6	3.0
$C_{17}H_{12}NO_4$[j]	$+H^+$	304.1555	304.1549	0.6	19.2
$C_{17}H_{23}NO_4$[k]	$+H^+$	306.1673	306.1705	-3.2	2.5

[a] Measured masses fell within 5 mmu of the calculated mass. [b]Compound formula is consistent with tropine, previously identified in *Datura* species[41]. [c]Compound formula is consistent with vanillin, previously identified in *Datura* species[43]. [d]Compound formula is consistent with scopoline, previously identified in *Datura* species[41]. [e]Compound formula is consistent with dihydroxytropane, previously identified in *Datura* species[41]. [f]Compound formula is consistent with hexose sugar, previously identified in *Datura* species[41]. [g]Compound formula is consistent with 3-tiglyloxytropane, previously identified in *Datura* species[41]. [h]Compound formula is consistent with atropine, previously identified in *Datura* species[41]. [i]Compound formula is consistent with linoleic acid, previously identified in *Datura* species[43]. [j]Compound formula is consistent with scopolamine, previously identified in *Datura* species[41]. [k]Compound formula is consistent with hydroxyhyoscyamine, previously identified in *Datura* species[41].

**Supplementary Table 5e.** DART-TOF-MS data for selected peaks in the spectrum of *H. niger* shown in Supplementary Figure 5.					
**Formula**	**Adduct**	**Measured**	**Calculated**	**Difference[a]**	**Abundance**
C_7H_{10}[b]	$+H^+$	95.0875	95.0861	1.4	1.7
C_7H_{10}[c]	$+NH_4^+$	112.1161	112.1126	3.5	2.6
$C_8H_{15}NO$[d]	$+H^+$	142.1254	142.1232	2.2	97.5
$C_7H_{13}NO_4$[e]	$+H^+$	176.0919	176.0923	-0.4	70.4
$C_6H_{12}O_6$[f]	$+NH_4^+$	198.0965	198.0978	-1.3	27.1
$C_{13}H_{21}NO_5$[g]	$+H^+$	272.1516	272.1498	1.8	3.8
$C_{18}H_{32}O_2$[h]	$+H^+$	281.2437	281.2481	-4.3	61.1
$C_{17}H_{23}NO_3$[i]	$+H^+$	290.1719	290.1756	-3.7	67.2
$C_{16}H_{19}NO_3$[j]	$+NH_4^+$	291.1750	291.1709	4.1	13.0
$C_{18}H_{32}O_2$[k]	$+NH_4^+$	298.2733	298.2756	-1.3	61.8
$C_{17}H_{12}NO_4$[l]	$+H^+$	304.1571	304.1549	2.2	80.8

[a] Measured masses fell within 5 mmu of the calculated mass.

Supplementary Tables 6a-6e. Mass spectral data associated with the positive ion DART-TOF-MS spectra of *E. bridgesiana*, *E. cineraria*, *E. globulus*, *E. citriodora*, and *E. polyanthemos* that are shown in Supplementary Figure 6. The observed molecular formulas are consistent with the tentative compound assignments listed.

Supplementary Table 6a. DART-TOF-MS data for selected peaks in the spectrum of *E. bridgesiana* shown in Supplementary Figure 6.

Formula	Adduct	Measured	Calculated	Difference[a]	Abundance
$C_{10}H_{16}$[b]	$+H^+$	137.1338	137.1330	0.8	92.9
$C_{10}H_{18}O$[c]	$+H^+$	155.1416	155.1436	-2.0	31.2
$C_{15}H_{24}$[d]	$+H^+$	205.1931	205.1956	-2.5	53.4
$C_{13}H_{14}O_5$[e]	$+H^+$	251.0876	251.0920	-4.4	90.3
$C_{18}H_{16}O_5$[f]	$+H^+$	313.1057	313.1076	1.9	32.8
$C_{19}H_{18}O_5$[g]	$+H^+$	327.1210	327.1232	-2.2	56.8
$C_{23}H_{30}O_5$[h]	$+H^+$	387.2179	387.2171	0.8	86.5
$C_{30}H_{46}O_2$[i]	$+H^+$	439.3544	439.3576	-3.2	56.5
—[j]		455.3334	—	—	100.0

[a]Measured masses fell within 5 mmu of the calculated mass. [b]Compound formula is consistent with previously identified monoterpenes found in *Eucalyptus* species, including camphene, pinene, limonene and thujene[45,46]. [c]Compound formula is consistent with eucalyptol, previously identified in *Eucalyptus* species[45,46]. [d]Compound formula is consistent with previously identified sesquiterpenes found in *Eucalyptus* species, including humulene, and caryophyllene[45,46]. [e]Compound formula is consistent with citrinin, previously identified in *Eucalyptus* species[45,46]. [f]Compound formula is consistent with isoflavone, previously identified in *Eucalyptus* species[45,46]. [g]Compound formula is consistent with eucalyptin, previously identified in *Eucalyptus* species[45,46]. [h-j] Unknown compounds

**Supplementary Table 6b.** DART-TOF-MS data for selected peaks in the spectrum of *E. cineraria* shown in Supplementary Figure 6.					
**Formula**	**Adduct**	**Measured**	**Calculated**	**Difference[a]**	**Abundance**
$C_{10}H_{16}$[b]	$+H^+$	137.1347	137.1330	1.7	100.0
$C_{10}H_{18}O$[c]	$+H^+$	155.1421	155.1436	-1.5	33.8
$C_{15}H_{24}$[d]	$+H^+$	205.1938	205.1956	-1.8	28.2
$C_{13}H_{14}O_5$[e]	$+H^+$	251.0917	251.0920	-0.3	23.1
$C_{19}H_{18}O_5$[f]	$+H^+$	327.1261	327.1232	-2.9	30.9
$C_{23}H_{30}O_5$[g]	$+H^+$	387.2203	387.2171	3.2	51.5
—[h]		455.3283	—	—	100.0

[a] Measured masses fell within 5 mmu of the calculated mass. [b]Compound formula is consistent with previously identified monoterpenes found in *Eucalyptus* species, including camphene, pinene, limonene and thujene[45,46]. [c]Compound formula is consistent with eucalyptol, previously identified in *Eucalyptus* species[45,46]. [d]Compound formula is consistent with previously identified sesquiterpenes found in *Eucalyptus* species, including humulene, and caryophyllene[45,46]. [e]Compound formula is consistent with citrinin, previously identified in *Eucalyptus* species[45,46]. [f]Compound formula is consistent with eucalyptin, previously identified in *Eucalyptus* species[45,46]. [g-h] Unknown compounds

**Supplementary Table 6c.** DART-TOF-MS data for selected peaks in the spectrum of *E. globulus* shown in Supplementary Figure 6.					
**Formula**	**Adduct**	**Measured**	**Calculated**	**Difference[a]**	**Abundance**
$C_{10}H_{16}$[b]	$+H^+$	137.1342	137.1330	1.2	73.8
$C_{10}H_{18}O$[c]	$+H^+$	155.1422	155.1436	-0.6	19.2
$C_{15}H_{24}$[d]	$+H^+$	205.1943	205.1956	-1.3	38.7
$C_{13}H_{14}O_5$[e]	$+H^+$	251.0927	251.0920	0.7	74.1
$C_{18}H_{16}O_5$[f]	$+H^+$	313.1076	313.1076	0.0	49.5
$C_{23}H_{30}O_5$[g]	$+H^+$	387.2202	387.2171	3.1	100.0
$C_{28}H_{38}O_5$[h]	$+H^+$	455.2848	455.2798	5.0	39.8

[a] Measured masses fell within 5 mmu of the calculated mass. [b]Compound formula is consistent with previously identified monoterpenes found in *Eucalyptus* species, including camphene, pinene, limonene and thujene[45,46]. [c]Compound formula is consistent with eucalyptol, previously identified in *Eucalyptus* species[45,46]. [d]Compound formula is consistent with previously identified sesquiterpenes found in *Eucalyptus* species, including humulene, and caryophyllene[45,46]. [e]Compound formula is consistent with citrinin, previously identified in *Eucalyptus* species[45,46]. [f]Compound formula is consistent with isoflavone, previously identified in *Eucalyptus* species[45,46]. [g] Unknown compound. [h]Compound formula is consistent with sesquiterpenes, phloroglucinol and dialdehyde diterpenes previously identified in *Eucalyptus* species[45,46].

**Supplementary Table 6d.** DART-TOF-MS data for selected peaks in the spectrum of *E. citriodora* shown in Supplementary Figure 6.					
**Formula**	**Adduct**	**Measured**	**Calculated**	**Difference[a]**	**Abundance**
$C_{10}H_{16}$[b]	$+H^+$	137.1376	137.1330	4.6	100.0
$C_{10}H_{18}O$[c]	$+H^+$	155.1450	155.1436	1.4	61.8
$C_{15}H_{24}$[d]	$+H^+$	205.1957	205.1956	0.1	10.7
$C_{30}H_{46}O_2$[e]	$+H^+$	439.3552	439.3576	-2.4	61.1

[a]Measured masses fell within 5 mmu of the calculated mass. [b]Compound formula is consistent with previously identified monoterpenes found in *Eucalyptus* species, including camphene, pinene, limonene and thujene[45,46]. [c]Compound formula is consistent with eucalyptol, previously identified in *Eucalyptus* species[45,46]. [d]Compound formula is consistent with previously identified sesquiterpenes found in *Eucalyptus* species, including humulene, and caryophyllene. [45,46] [e]Unknown compound.

**Supplementary Table 6e.** DART-TOF-MS data for selected peaks in the spectrum of *E. polyanthemos* shown in Supplementary Figure 6.					
**Formula**	**Adduct**	**Measured**	**Calculated**	**Difference**[a]	**Abundance**
C_7H_{11}[b]	$+H^+$	95.0921	95.0861	6.0	81.0
$C_{10}H_{16}$[c]	$+H^+$	137.1361	137.1330	3.1	100.0
$C_{15}H_{24}$[d]	$+H^+$	205.1957	205.1956	0.1	19.4
$C_{13}H_{14}O_5$[e]	$+H^+$	251.0932	251.0920	1.2	41.5
$C_{23}H_{30}O_5$[f]	$+H^+$	387.2193	387.2171	2.2	30.0
$C_{28}H_{38}O_5$[g]	$+H^+$	455.2855	255.2798	5.7	
$C_{30}H_{48}O_3$[h]	$+H^+$	457.3675	457.3682	-0.7	22.1

[a]With the exception of the sesquiterpene fragment, measured masses fell within 5 mmu of the calculated masses. [b]Compound formula is consistent with a sesquiterpene fragment. [c]Compound formula is consistent with previously identified monoterpenes found in *Eucalyptus* species, including camphene, pinene, limonene and thujene[45,46]. [d]Compound formula is consistent with previously identified sesquiterpenes found in *Eucalyptus* species, including humulene, and caryophyllene[45,46]. [e]Compound formula is consistent with citrinin, previously identified in *Eucalyptus* species[45,46]. [f]Unknown compound. [g]Compound formula is consistent with sesquiterpenes, phloroglucinol and dialdehyde diterpenes previously identified in *Eucalyptus* species[45,46]. [h]Compound formula is consistent with betulinic, oleanolic or ursolic acid, previously identified in *Eucalyptus* species[47].
